# Supplementary material for: Bridging the knowledge gap on the evolution of the Asian monsoon during 26–16 Ma
Source: Innovation (Camb). 2021 Apr 29;2(2):100110. doi: 10.1016/j.xinn.2021.100110 (PMC8454623; doi:10.1016/j.xinn.2021.100110)
Supplement: Document S1. Supplemental materials and methods, Figures S1–S7, and Tables S1 and S3 [file mmc1.pdf]

The Innovation, Volume *2*

Supplemental Information

Bridging the knowledge gap on the evolution of the Asian monsoon during 26–16 Ma

Gan Xie, Jin-Feng Li, Shi-Qi Wang, Yi-Feng Yao, Bin Sun, David K. Ferguson, Cheng-Sen Li, Tao Deng, Xiao-Dong Liu, and Yu-Fei Wang

This PDF file includes:

Supplemental Materials and Methods
Supplemental References 31-50
Figures S1 to S7
Table S1 and S3

Supplemental Materials and methods

Geological setting. The study section is the Dingqing Formation located in the Lunpori Mountains of the Lunpola Basin, Bange County, central Tibet (31°56′-31°57′ N, 89°47′-89°49′ E, 4650 m.a.s.l.) (see Fig. S1). This section is divided into 28 layers from bottom to top. For descriptions of the lithostratigraphy, see Deng et al.[13, 31]. Ninety-nine palynological samples were collected from layers 1 to 17 in this section (see Fig. S2).

Dating. Here, we adopt the concept of the stratigraphic sequence and identifying its age by Deng et al.[31]. Deng et al. reported a fossil rhino (*Plesiaceratherium*) from layer 17 in this section[13], which is the uppermost layer of sampling in this work. The genus *Plesiaceratherium* occurred worldwide in the late Early Miocene between the cold events Mi-1b at 17.8 Ma and Mi-2 at 16 Ma[13]. Because *Plesiaceratherium* was found together with a fossil hyena (*Percrocuta*) in Jiulongkou, Ci County, Hebei Province, China, which appeared later than the *Plesiaceratherium* in geologic history[13], we speculate that *Plesiaceratherium* appeared in China at ~16 Ma. At the same time, layer 7, which is a bentonite layer, was found to be 20.6 to 20.7 ± 0.1 Ma by LA-ICP-MS zircon U-Pb dating[32], while layer 2 was calibrated as 25.5±0.5 Ma by Su et al.[23] based on magnetostratigraphic data[33] and U-Pb dating[34]. Thus, we estimate the sedimentation rate of the upper part (layers 1-7) of this section as ~75 m/Ma and the bottom part

(layers 8-17) of this section as ~107 m/Ma; therefore, we can calculate the age of each collected sample (see Table S1). According to our calculations, the ages of our samples ranged from 26 to 16 Ma, covering the Late Oligocene to Early Miocene.

Pollen analysis. All the samples were treated with a heavy-liquid separation[35] method (density: 2.0 g/ml) to extract the pollen. The pollen and spores were identified as modern taxa using a Leica DM 2500 light microscope and by referring to the palynological literature[36-39] and monographs (Fig. S3). Abundant pollen and spores were found in 66 samples (56-1559 grains per slide), and over 300 grains were counted in most of these samples (Tables S2, S3). Those pollen and spores that could not be referred to modern taxa were regarded as an unknown type. Another 33 samples (Sample 12, 23-26, 28-37, 39, 40, 42, 44, 48, 50, 51, 54, 55, 60, 63, 65, 68, 81, 84, 94, 97 and 98) that preserved only a few pollen grains (2-26 grains per slide) were not counted or used in the reconstruction of the climatic data.

Paleo-climatic data. We applied the coexistence approach (CoA)[12] to reconstruct the climatic data in the Lunpola Basin during the Late Oligocene to Early Miocene. Four climate parameters were obtained (Table S4), i.e., MAT, MAP, 3HMP, and 3LMP. The modern distributions of pollen taxa in China[40] and the meteorological data within these distributions areas[15] were extracted to calibrate the climate interval of each parameter and the coexistence interval.

The detailed steps of calculating the climate parameters by the CoA are explained below. First, we ascertained which plant taxa lived in the Lunpola Basin, central Tibet,

during ~26-16 Ma based on the pollen extracted from sediments (see Table S2). Second, we obtained the distribution of each taxon in modern China by consulting Wu and Ding[40]. Third, we ascertained the climatic data for the regions in which each taxon lives from the Surface Meteorological Data of China (1951-1980)[15]. Next, we superimposed the climatic intervals of all the distribution areas of each taxon to constrain the range of the above four climate parameters. Finally, we calibrated the coincidence intervals of these climate parameters for all plant taxa (e.g., Fig. S4).

Paleo-monsoon. Here, we introduce the definition of the modern Asian monsoon[2] to calibrate the Asian monsoon during ca. 26-16 Ma. In the modern Asian monsoon domains, the annual range of precipitation (AR, summer minus winter) should be greater than 180 mm, the proportion of summer (June to August) precipitation in the annual precipitation should be greater than 35%, while larger values of AR correspond to stronger Asian monsoons[2]. We used the 3HMP and 3LMP to depict summer and winter precipitation, respectively; thus, the intensity of the Asian monsoon, as estimated by AR, could be described as 3HMP minus 3LMP. We also conducted spectral analysis to evaluate the frequency of the Asian monsoon intensity using the paleontological statistics software PAST 3.

Paleo-climatic modeling. The climate model used in this study was the Fast Met Office/UK Universities Simulator, which is a coupled atmosphere-ocean general circulation model (FAMOUS AOGCM)[41, 42]. The spatial resolution of the atmospheric component of FAMOUS was 5° × 7.5°, with 11 vertical layers, while the spatial resolution of the ocean component was 2.5° × 3.75°, with 20 vertical levels. The

atmospheric and oceanic components were coupled once every day, with no adjustments for fluxes. In the numerical experiment for the Late Oligocene (~25 Ma), we used a land-ocean configuration and topography that were reconstructed based on geological evidence. The experiment was run for 1000 years, and our analyses were based on the averages from the last 100 years. The land-ocean distributions during geological times were mainly based on the data from the GPlates database, an open-source software for the reconstruction of plate motions during the geologic time periods[43]. For Asia and Europe, the coastlines were modified according to varying regional geological evidence (e.g., Popov et al.[44]). The paleotopography and paleobathymetry were established based on previous reconstructions[45, 46] but revised using a large amount of published paleoelevation data (ref. Liu et al.[47]). The paleotopography of the Tibetan Plateau was reconstructed using paleoelevation data from various sources (e.g., Wang et al.[48]). Changes in paleolatitude were also considered for the Tibetan Plateau[49, 50]. More details on the experimental design and the boundary conditions related to the land-ocean configuration and the plateau topography can be found in Liu et al.[8, 47].

Supplemental References

31. Deng, T., Wang, X., Wu, F., Wang, Y., Li, Q., Wang, S. and Hou, S. Implications of vertebrate fossils for paleo-elevations of the Tibetan Plateau. *Global and planetary change*, (2019).
32. Mao, Z., Meng, Q., Fang, X., Zhang, T., Wu, F., Yang, Y., Zhang, W., Zan, J. and Tan, M. Recognition of tuffs in the middle-upper Dingqinghu Fm., Lunpola Basin, central Tibetan Plateau: Constraints on stratigraphic age and implications for paleoclimate. *Palaeogeography, Palaeoclimatology, Palaeoecology* **525**, 44-56 (2019).
33. Sun, J., Xu, Q., Liu, W., Zhang, Z., Xue, L. and Zhao, P. Palynological evidence for the latest Oligocene-early Miocene paleoelevation estimate in the Lunpola Basin, central Tibet. *Palaeogeography, Palaeoclimatology, Palaeoecology* **399**, 21-30 (2014).
34. He, H., Sun, J., Li, Q. and Zhu, R. New age determination of the Cenozoic Lunpola Basin,central Tibet. *Geological Magazine* **149**(1), 141-145 (2012).
35. Li, X. and Du, N. The acid-alkali-free analysis of Quaternary pollen. *Acta Botanica Sinica* **41**(7), 782-784 (1999).
36. Institute of Botany, C.A.S., *Sporae Pteridophytorum Sinicorum*. (Science Press, Beijing, 1976).
37. Institute of Botany, C.A.S., *Angiosperm Pollen Flora of Tropic and Subtropic China*. (Science Press, Beijing, 1982).
38. Wang, F.-X., Chien, N.-F., Zhang, Y.-L. and Yang, H.-Q., *Pollen Flora of China*. (Science Press, Beijing, ed. Second Edition, 1991).
39. Song, Z., *Fossil spore and pollen of China: The Late Cretaceous and Tertiary spore and pollen*. (Science Press, Beijing, 1999).
40. Wu, Z. and Ding, T., *Seed plants of China*. (Yunnan Science and Technology Press, Kunming, Yunnan, 1999).
41. Jones, C., Gregory, J., Thorpe, R., Cox, P., Murphy, J., Sexton, D. and Valdes, P. Systematic optimisation and climate simulation of FAMOUS, a fast version of HadCM3. *Climate Dynamics* **25**(2-3), 189-204 (2005).
42. Smith, R.S., Gregory, J.M. and Osprey, A. A description of the FAMOUS (version XDBUA) climate model and control run. *Geoscientific Model Development* **1**(1), 53-68 (2008).
43. Gurnis, M., Turner, M., Zahirovic, S., DiCaprio, L., Spasojevic, S., Müller, R.D., Boyden, J., Seton, M., Manea, V.C. and Bower, D.J. Plate tectonic reconstructions with continuously closing plates. *Computers & Geosciences* **38**(1), 35-42 (2012).
44. Popov, S.V., Shcherba, I.G., Ilyina, L.B., Nevesskaya, L.A., Paramonova, N.P., Khondkarian, S.O. and Magyar, I. Late Miocene to Pliocene palaeogeography of the Paratethys and its relation to the Mediterranean. *Palaeogeography, Palaeoclimatology, Palaeoecology* **238**(1-4), 91-106 (2006).
45. Herold, N., Seton, M., Müller, R., You, Y. and Huber, M. Middle Miocene tectonic boundary conditions for use in climate models. *Geochemistry, Geophysics, Geosystems* **9**(10), (2008).
46. Huber, M. and Goldner, A. Eocene monsoons. *Journal of Asian Earth Sciences* **44**, 3-23 (2012).
47. Liu, X., Dong, B., Yin, Z.Y., Smith, R.S. and Guo, Q. Continental drift and plateau uplift control origination and evolution of Asian and Australian monsoons. *Scientific Reports* **7**, 40344 (2017).
48. Wang, C., Dai, J., Zhao, X., Li, Y., Graham, S.A., He, D., Bo, R. and Meng, J. Outward-growth of the Tibetan Plateau during the Cenozoic: A review. *Tectonophysics* **621**(Complete), 1-43

(2014).
49. Chatterjee, S., Goswami, A. and Scotese, C.R. The longest voyage: tectonic, magmatic, and paleoclimatic evolution of the Indian plate during its northward flight from Gondwana to Asia. *Gondwana Research* **23**(1), 238-267 (2013).
50. Wei, H.H., Meng, Q.R., Ding, L. and Li, Z.Y. Tertiary evolution of the western Tarim basin, northwest China: A tectono-sedimentary response to northward indentation of the Pamir salient. *Tectonics* **32**(3), 558-575 (2013).

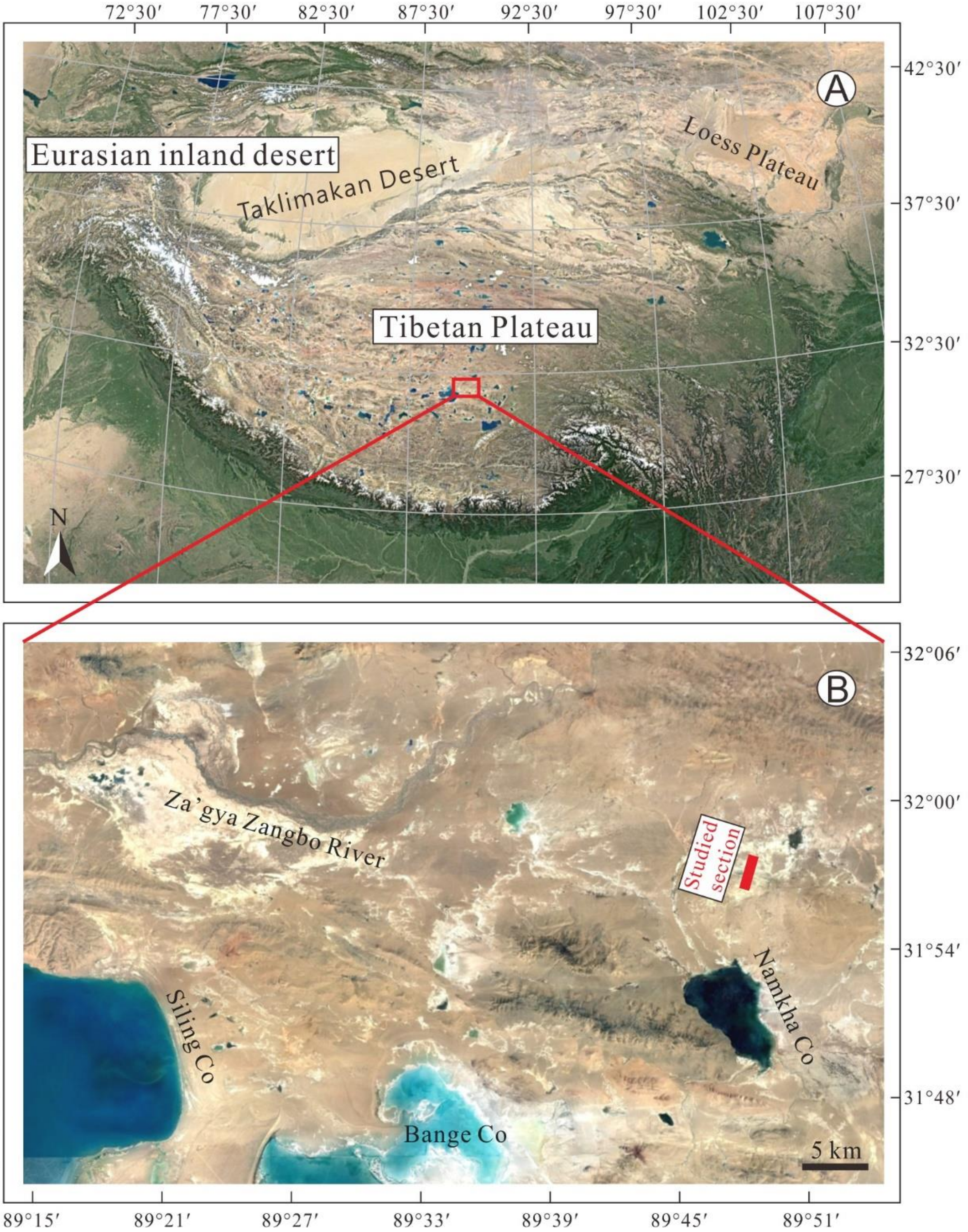

Fig. S1. Maps showing the position of the Lunpola locality (B) in central Tibet (A), modified from Google Earth images.

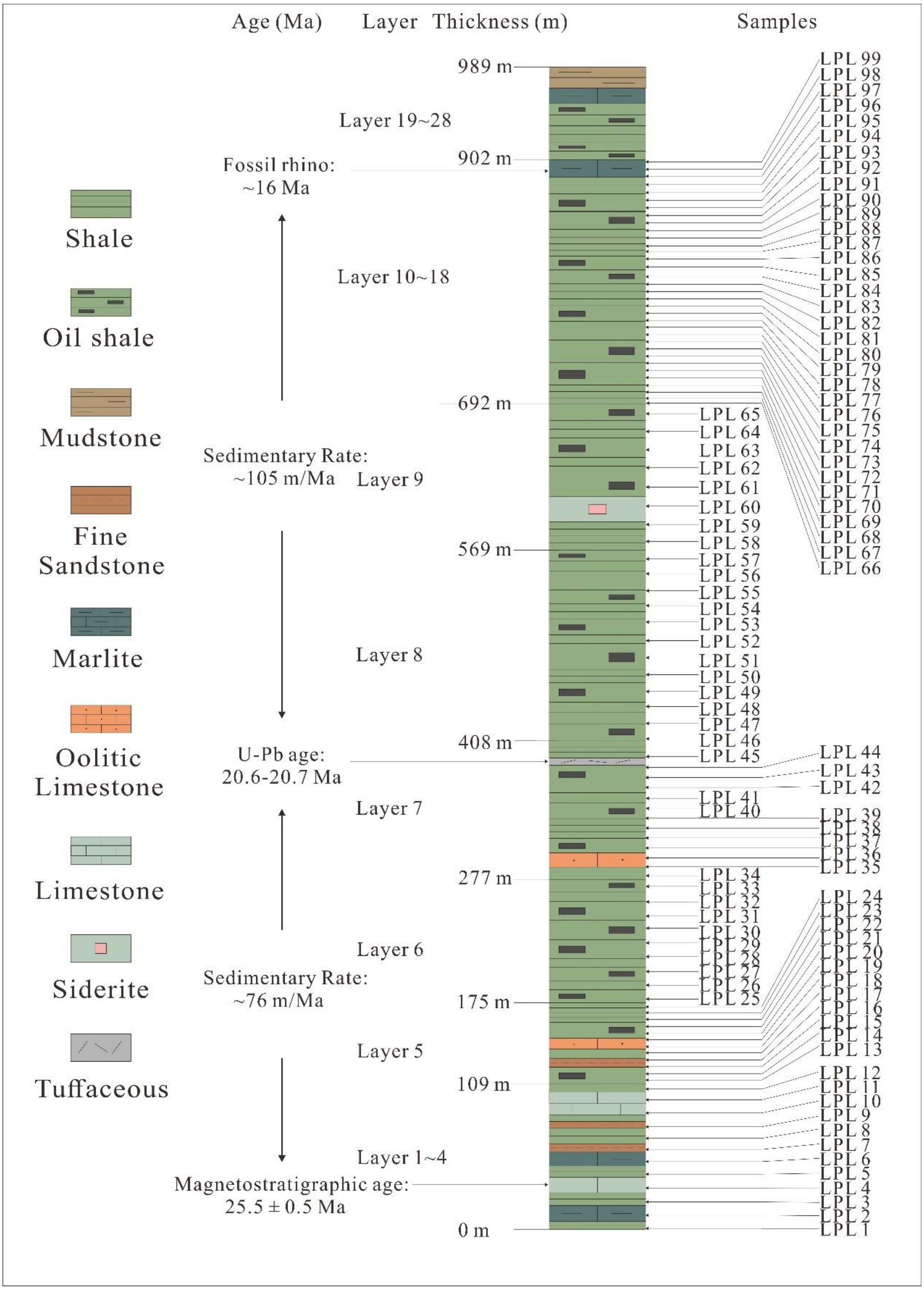

Fig. S2. Measured stratigraphical sequence of the Dingqing Formation section in the Lunpola Basin, central Tibet.

Age sources: fossil rhino, Deng et al.[13]; U-Pb age, Mao et al.[32]; magnetostratigraphic age, Su et al.[23].

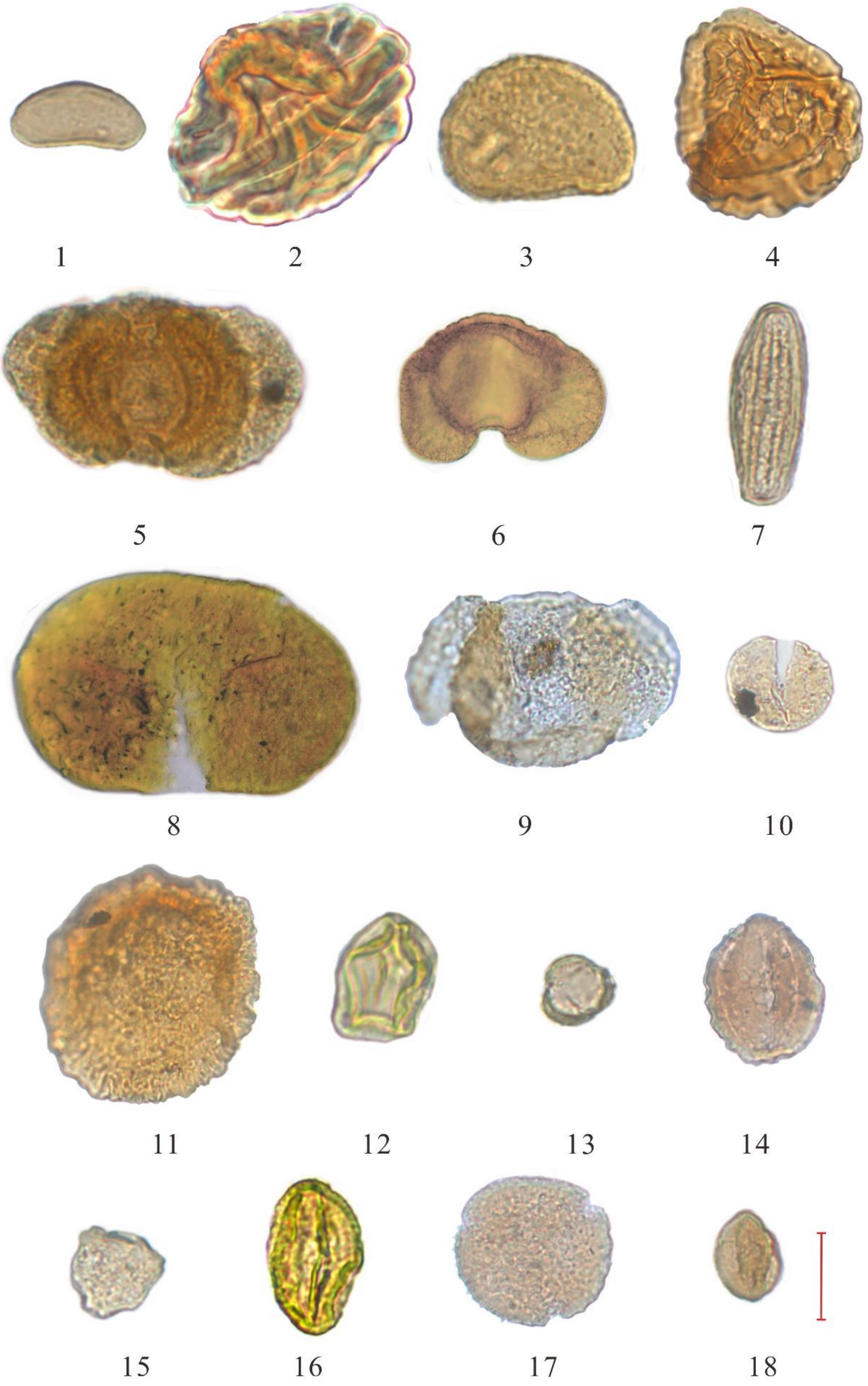
1
2
3
4
5
6
7
8
9
10
11
12
13
14
15
16
17
18

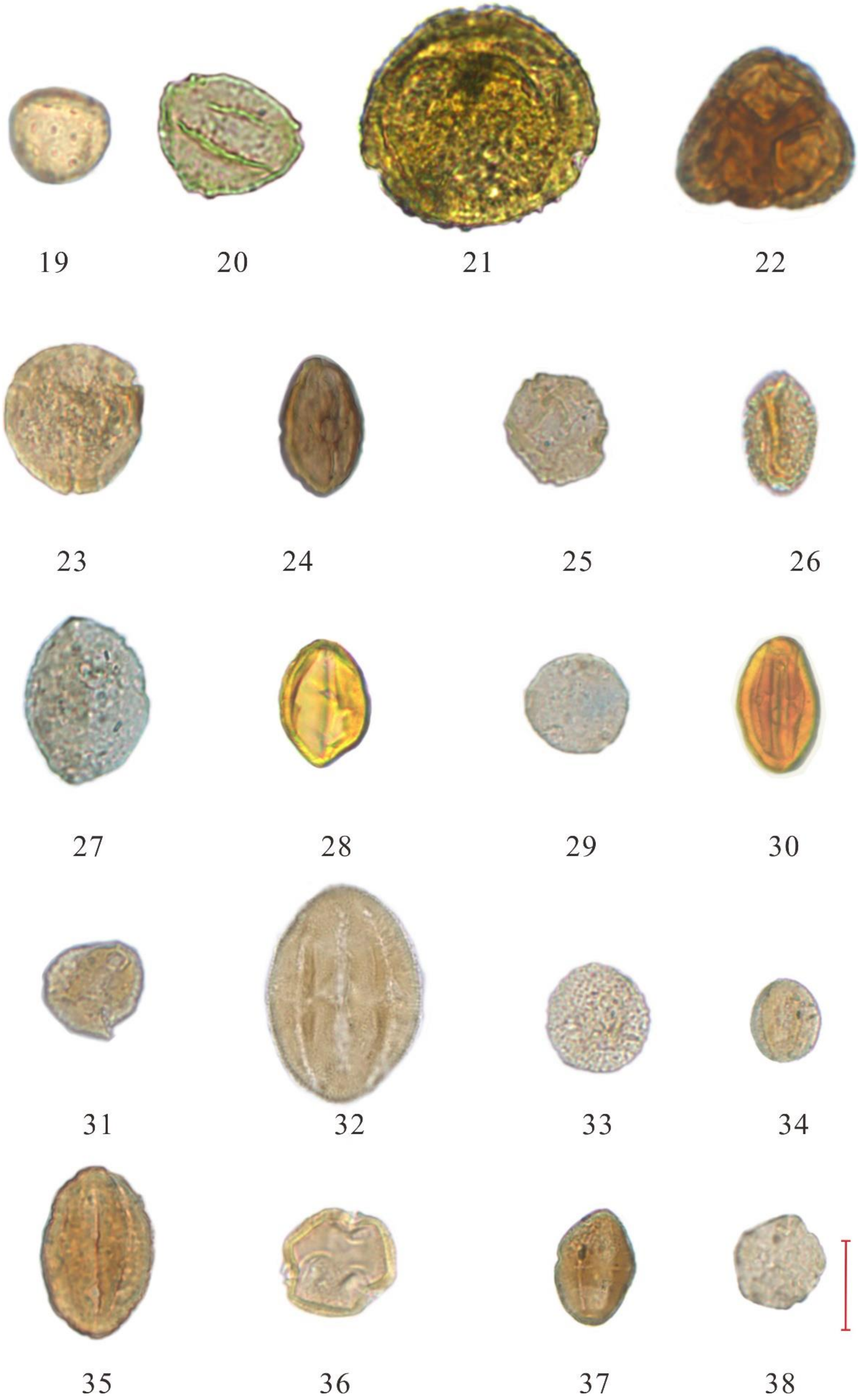

Fig. S3. Palynomorphs recovered from the Dingqing Formation. Scale bars: 20 μm
1. Athyriaceae; 2. Parkeriaceae; 3. Polypodiaceae; 4. Pteridaceae; 5. *Abies*; 6. *Cedrus*; 7. *Ephedra*; 8. *Picea*; 9. *Pinus*; 10. Taxodiaceae; 11. *Tsuga*; 12. *Alnus*; 13. *Artemisia*;

14. Asteraceae; 15. *Betula*; 16. Brassicaceae; 17. Caprifoliaceae; 18. *Castanopsis*; 19. Chenopodiaceae; 20. *Corylus*; 21. Dipsacaceae; 22. Ericaceae; 23. Euphorbiaceae; 24. Fabaceae; 25. *Juglans*; 26. Liliaceae; 27. Magnoliaceae; 28. Meliaceae; 29. Moraceae; 30. *Nitraria*; 31. Poaceae; 32. Polygonaceae; 33. Potamogetonaceae; 34. *Quercus*; 35. Ranunculaceae; 36. Rosaceae; 37. Rutaceae; 38. *Ulmus*.

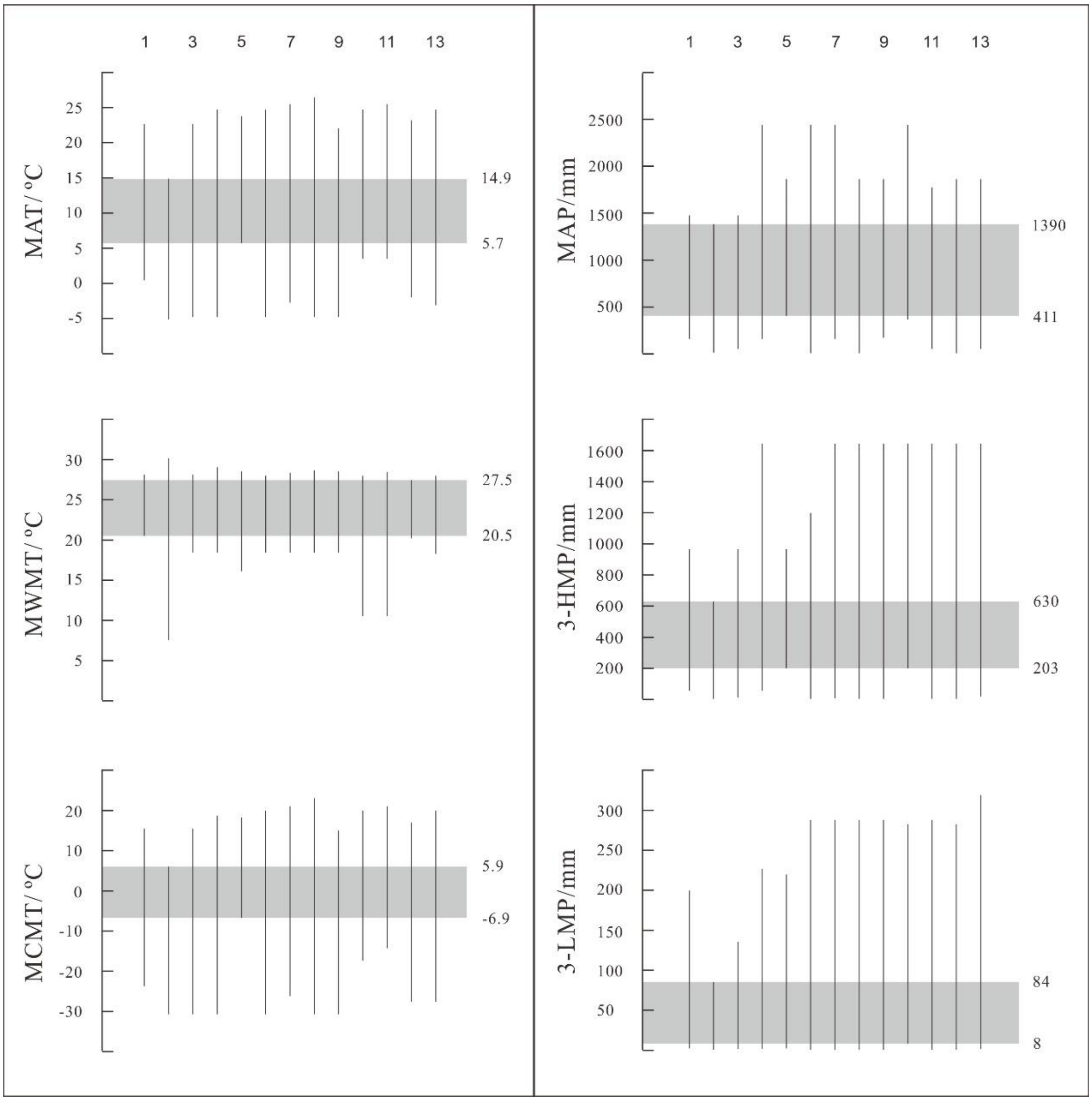

Fig. S4. The coexistence interval of the climatic parameters of the Sample No. 1 palynomorphs in Lunpola Basin

1. *Abies*; 2. *Ephedra*; 3. *Picea*; 4. *Pinus*; 5. *Tsuga*; 6. Chenopodiaceae; 7. Euphorbiaceae; 8. Fabaceae; 9. Lamiaceae; 10. Magnoliaceae; 11. Polygonaceae; 12. Ranunculaceae; 13. Rutaceae.

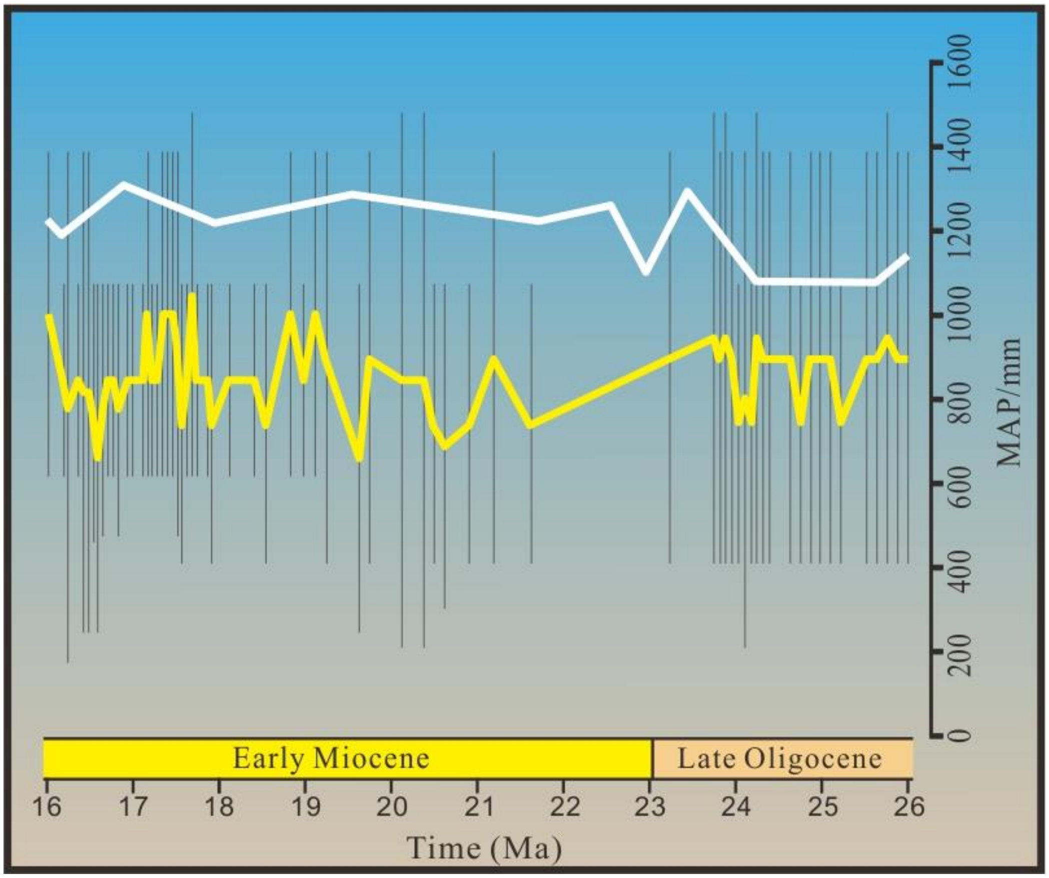

Fig. S5. MAP curves for central Tibet and central Europe ca. 26-16 Ma.
The yellow line shows the MAP (mm) in central Tibet, which was derived from this study; the white line indicates the MAP (mm) in central Europe, which was modified from Mosbrugger et al.[16].

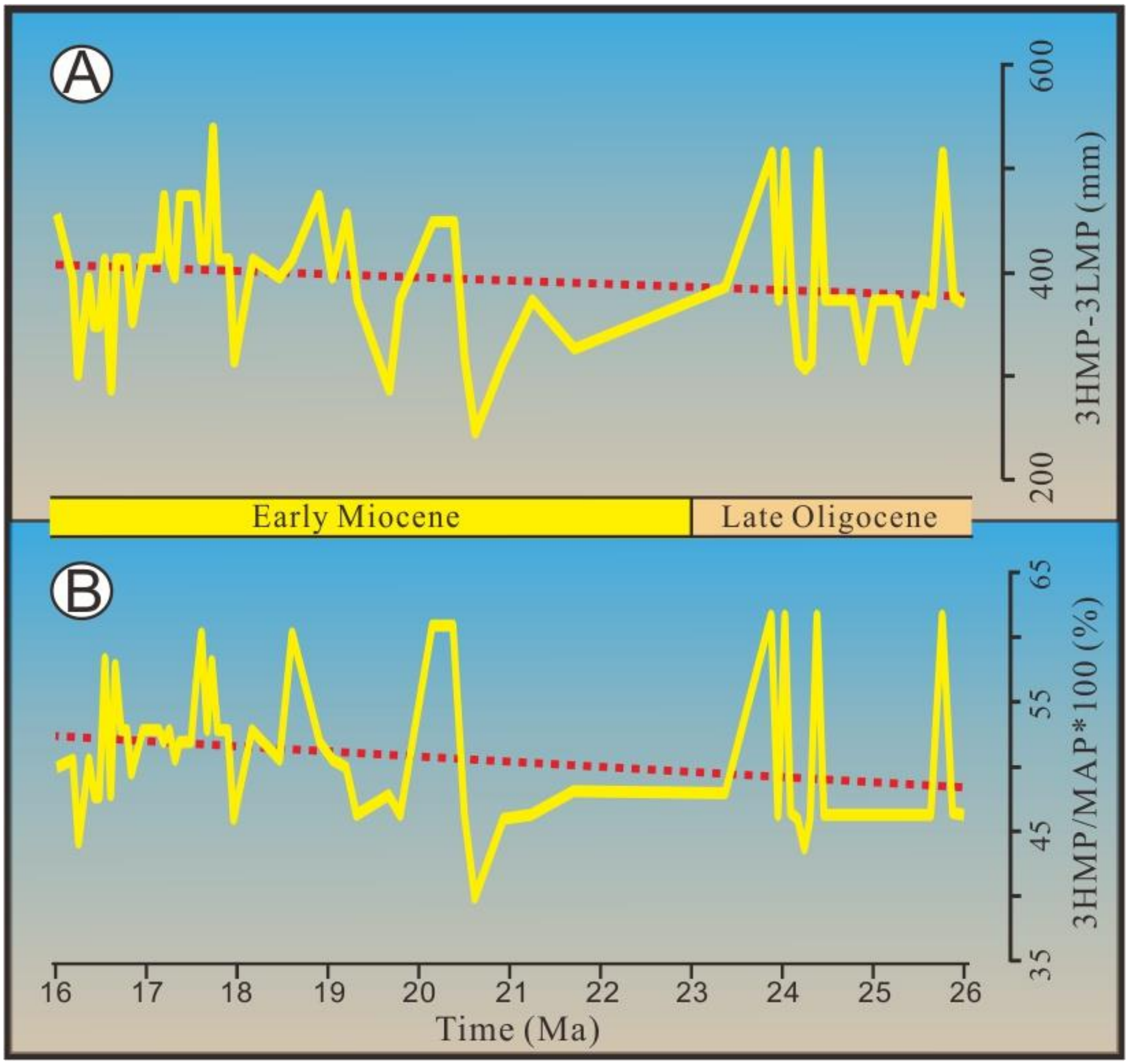

Fig. S6. Maps showing the fluctuations in the Asian monsoon in the Lunpola Basin, central Tibet during 26-16 Ma.

(A) the annual range of precipitation (AR, 3HMP minus 3LMP, mm); (B) the proportion of summer precipitation in the annual precipitation (%). The yellow line indicates the fluctuations; the red dotted line indicates the general trend.

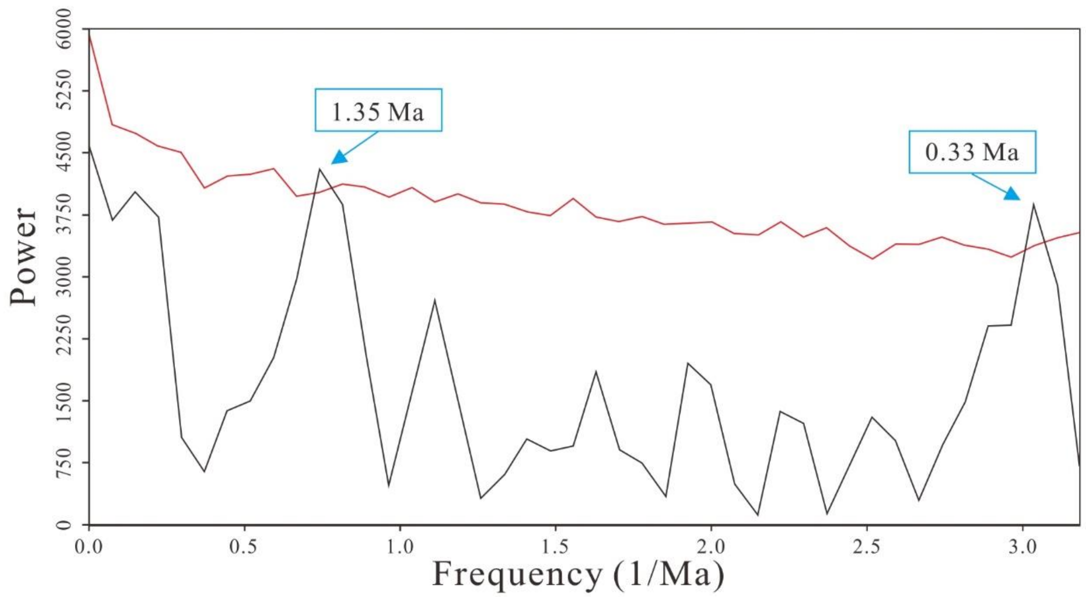

Fig. S7. Results of REDFIT spectral analysis.
The periodicity (from left to right): 1.35 Ma, 0.33 Ma; red line: significance line at the 95% confidence level for a Monte Carlo test

Table S1 Depth and calculated age of each sample

Layer	Thickness (m)	Amount of Samples	Sample	Depth (m)	Increasing of depth (m/sample)	Sedimentary Rate (m/Ma)	Increasing of time (Ma/sample)	Age (Ma)	Error range (Ma)
10-18	210	34	99	902.0	6.18	107	0.06	16.00	/
			98	895.8				16.06	± 0.1
			97	889.6				16.12	
			96	883.5				16.17	
			95	877.3				16.23	
			94	871.1				16.29	
			93	864.9				16.35	
			92	858.8				16.40	
			91	852.6				16.46	
			90	846.4				16.52	
			89	840.2				16.58	
			88	834.1				16.63	
			87	827.9				16.69	
			86	821.7				16.75	
			85	815.5				16.81	
			84	809.4				16.86	
			83	803.2				16.92	
			82	797.0				16.98	
			81	790.8				17.04	
			80	784.6				17.09	
			79	778.5				17.15	
			78	772.3				17.21	
			77	766.1				17.27	
			76	759.9				17.32	
			75	753.8				17.38	
			74	747.6				17.44	
			73	741.4				17.50	
			72	735.2				17.55	
			71	729.1				17.61	
			70	722.9				17.67	
			69	716.7				17.73	
			68	710.5				17.78	
			67	704.4				17.84	
			66	698.2				17.90	
9	123	8	65	692.0	15.38		0.14	17.96	
			64	676.6				18.10	
			63	661.3				18.24	
			62	645.9				18.38	
			61	630.5				18.53	
			60	615.1				18.67	
			59	599.8				18.81	

			58	584.4				18.96	
8	161	12	57	569.0	13.42		0.12	19.10	
			56	555.6				19.23	
			55	542.2				19.35	
			54	528.8				19.48	
			53	515.3				19.60	
			52	501.9				19.73	
			51	488.5				19.85	
			50	475.1				19.98	
			49	461.7				20.10	
			48	448.3				20.23	
			47	434.8				20.35	
			46	421.4				20.48	
7	131	12	45	408.0	10.92	75	0.15	20.60	± 0.1
			44	392.5				20.75	± 0.6
			43	381.6				20.89	
			42	370.7				21.04	
			41	359.8				21.18	
			40	348.8				21.33	
			39	337.9				21.47	
			38	327.0				21.62	
			37	316.1				21.77	
			36	305.2				21.91	
			35	294.3				22.06	
			34	283.3				22.20	
6	102	9	33	272.4	11.33		0.15	22.35	
			32	261.1				22.51	
			31	249.8				22.66	
			30	238.4				22.81	
			29	227.1				22.96	
			28	215.8				23.11	
			27	204.4				23.26	
			26	193.1				23.41	
			25	181.8				23.56	
5	66	12	24	170.4	5.50		0.07	23.64	
			23	164.9				23.71	
			22	159.4				23.78	
			21	153.9				23.86	
			20	148.4				23.93	
			19	142.9				24.00	
			18	137.4				24.08	
			17	131.9				24.15	
			16	126.4				24.22	
			15	120.9				24.30	
			14	115.4				24.37	

			13	109.9				24.44	
1-4	109	12	12	104.4	9.08		0.12	24.57	
			11	95.3				24.69	
			10	86.3				24.81	
			9	77.2				24.93	
			8	68.1				25.05	
			7	59.0				25.17	
			6	49.9				25.29	
			5	40.8				25.50	± 0.5
			4	31.8				25.62	± 0.6
			3	22.7				25.74	
			2	13.6				25.86	
			1	4.5				25.98	

Table S3. The palynomorph relative abundance in the Lunbori section

Palynomorph	RA(%)
Abies	4.4
Cedrus	0.5
Ephedra	3.0
Picea	37.0
Pinus	19.2
Taxodiaceae	/
Tsuga	0.9
Alnus	0.3
Artemisia	0.2
Asteraceae	1.0
Betula	0.8
Brassicaceae	/
Caprifoliaceae	0.1
Castanopsis	1.2
Chenopodiaceae	2.1
Corylus	/
Dipsacaceae	/
Ericaceae	0.1
Euphorbiaceae	0.2
Fabaceae	0.2
Fagaceae	1.3

Palynomorph	RA(%)
Juglans	0.1
Lamiaceae	0.6
Liliaceae	/
Magnoliaceae	/
Meliaceae	0.2
Moraceae	/
Nitraria	1.9
Poaceae	/
Polygonaceae	0.7
Potamogetonaceae	0.3
Quercus	0.4
Ranunculaceae	1.3
Rosaceae	0.4
Rutaceae	0.9
Ulmus	0.1
Athyriaceae	2.3
Parkeriaceae	0.1
Polypodiaceae	2.9
Pteridaceae	2.8
Unknown	12.4
/: less than 0.1%	
