# Supplementary material for: Bridging the knowledge gap on the evolution of the Asian monsoon during 26–16 Ma
Source: Innovation (Camb). 2021 Apr 29;2(2):100110. doi: 10.1016/j.xinn.2021.100110 (PMC8454623; doi:10.1016/j.xinn.2021.100110)
Supplement: Document S2. Article plus supplemental information [file mmc4.pdf]

Report

Bridging the knowledge gap on the evolution of the Asian monsoon during 26–16 Ma

Gan Xie,[1,6,7] Jin-Feng Li,[1,7] Shi-Qi Wang,[2,6,7] Yi-Feng Yao,[1] Bin Sun,[1] David K. Ferguson,[5] Cheng-Sen Li,[1] Tao Deng,[2,6,*] Xiao-Dong Liu,[3,4,*] and Yu-Fei Wang[1,6,*]
*Correspondence: dengtao@ivpp.ac.cn (T.D.); liuxd@loess.llqg.ac.cn (X.-D.L.); wangyf@ibcas.ac.cn (Y.-F.W.)
Received: October 14, 2020; Accepted: April 26, 2021; Published Online: April 28, 2021; https://doi.org/10.1016/j.xinn.2021.100110
© 2021 The Author(s). This is an open access article under the CC BY license (http://creativecommons.org/licenses/by/4.0/).

Graphical abstract

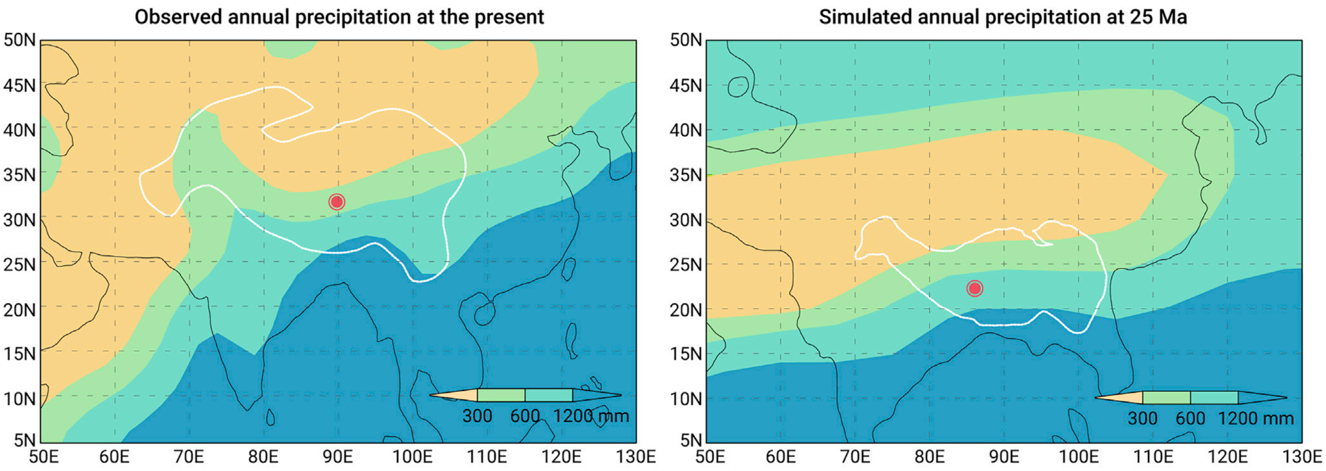

Public summary

- To reconstruct the precipitation changes in central Tibet during 26-16 Ma
- To depict the early evolution of Asian monsoon using the modern monsoon definition
- To explain the occurrence of the paleo-monsoon by paleoclimate modeling
- To reveal that long-period orbital forcings is responsible for the monsoon evolution

Report

Bridging the knowledge gap on the evolution of the Asian monsoon during 26–16 Ma

Gan Xie,[1,6,7] Jin-Feng Li,[1,7] Shi-Qi Wang,[2,6,7] Yi-Feng Yao,[1] Bin Sun,[1] David K. Ferguson,[5] Cheng-Sen Li,[1] Tao Deng,[2,6,*] Xiao-Dong Liu,[3,4,*] and Yu-Fei Wang[1,6,*]

[1]State Key Laboratory of Systematic and Evolutionary Botany, Institute of Botany, Chinese Academy of Sciences, Beijing 100093, China
[2]Institute of Vertebrate Paleontology and Paleoanthropology, Chinese Academy of Sciences, Beijing 100044, China
[3]State Key Laboratory of Loess and Quaternary Geology, Institute of Earth Environment, Chinese Academy of Sciences, Xi'an 710075, China
[4]Center for Excellence in Tibetan Plateau Earth Sciences, Chinese Academy of Sciences, Beijing 100101, China
[5]Department of Paleontology, University of Vienna, 1090 Vienna, Austria
[6]University of Chinese Academy of Sciences, Beijing 100049, China
[7]These authors contributed equally
*Correspondence: dengtao@ivpp.ac.cn (T.D.); liuxd@loess.llqg.ac.cn (X.-D.L.); wangyf@ibcas.ac.cn (Y.-F.W.)
Received: October 14, 2020; Accepted: April 26, 2021; Published Online: April 28, 2021; https://doi.org/10.1016/j.xinn.2021.100110
© 2021 The Author(s). This is an open access article under the CC BY license (http://creativecommons.org/licenses/by/4.0/).
Citation: Xie G., Li J.-F., Wang S.-Q., et al., (2021). Bridging the knowledge gap on the evolution of the Asian monsoon during 26–16 Ma. The Innovation **2(2)**, 100110.

The evolution of the Asian monsoon from the Late Oligocene to the Early Miocene is poorly understood. Here, we first reconstruct the precipitation data of central Tibet during 26–16 million years ago (Ma), applying the coexistence approach to sedimentary pollen data, and detect an intensified Asian monsoon with ~1.35 Ma and ~0.33 Ma cycles. Paleoclimate modeling is used to show the importance of paleogeographic location in the development of the paleomonsoon. In addition, the results of spectral analysis suggest that the fluctuations in the Asian monsoon during 26–16 Ma can be attributed to the long-period cyclicities in obliquity (~1.2 Ma). These findings provide climate data that can be used to understand the Asian monsoon evolution during the Late Oligocene to Early Miocene and highlight the effects of paleogeographic patterns and long-period orbital forcings on the tectonic-scale evolution of the Asian monsoon.

Keywords: **central Tibetan Plateau; Lunpola Basin; precipitation; Asian monsoon; Oligocene to Miocene; climate change**

INTRODUCTION

As an important part of global atmospheric circulation, the Asian monsoon greatly affects the ecological systems and social economies in Asia.[1] The domains of the modern Asian monsoon, including those of both the East Asian and South Asian monsoons, cover most of East Asia and almost all of South and Southeast Asia.[2] The Asian monsoon dominates the climate and supplies precipitation for the sustainable development of the ecosystems and populations.[3]

Currently, an increasing number of researchers are involved in identifying the conditions that led to the initial onset and development of the Asian monsoon. However, the evolution and related driving forces of the Asian monsoon are still inadequately understood. For example, some authors have proposed that the East Asian monsoon originated in the Late Eocene[4] under the influence of a high atmospheric CO_2 content[5] identified by models, and likely reached inner Asia during the Early Oligocene, as suggested by pollen assemblages.[6] Others have speculated that the modern East Asian monsoon was initiated in the Miocene and was triggered by the uplift of the Tibetan Plateau, based on geological evidence from loess deposits[7] and models.[8] The South Asian monsoon seems to have appeared in the Eocene,[9] with controversy regarding its driving force. The thermal effects[10] and dynamic consequences[11] resulting from the uplift of the Tibetan Plateau and changes in the paleogeography[8] were interpreted as the potential driving forces, based on different models.

The lack of paleoclimatic data for the regions and time intervals of interest prevent us from understanding the evolution and related driving forces of the Asian monsoon from the Late Eocene to the Miocene. To provide climate data and explore the evolution of the Asian monsoon, in this study, we (1) provide temperature and precipitation data for the Lunpola Basin, central Tibet, during ca. 26–16 million years ago (Ma) by applying a coexistence approach[12] to pollen data, (2) detect an Asian paleomonsoon that was delineated by the difference between the mean of the three consecutive highest monthly precipitations (3HMP) and the mean of the three consecutive lowest monthly precipitations (3LMP), (3) inspect the fluctuation cycles of the Asian paleomonsoon with spectral analysis, (4) model the paleogeography of the Pan-Tibetan Plateau and the monsoonal precipitation at 25 Ma, and (5) explore the potential driving forces of the Asian paleomonsoon.

RESULTS

The Dingqing Formation, which has yielded numerous animal and plant fossils, consists of a series of continuous lacustrine deposits in the Lunpola Basin in central Tibet that have been dated as ranging from the Late Oligocene to the Early Miocene.[13,14] The studied section (31°56′–31°57′ N, 89°47′–89°49′ E, 4,650 masl) is located on Lunbori Mountain (Figure S1), which dates back 26–16 Ma, allowing us to trace the early evolution of the Asian monsoon.

Ninety-nine palynological samples were collected from this section (Figure S2 and Table S1), among which 66 had an abundance of pollen grains. The pollen assemblages identified from these 66 samples yielded 40 palynomorphs that were assigned to 32 families and 15 genera (note: some palynomorphs were only identified to the family level), including 25 families and nine genera of angiosperms, three families and six genera of gymnosperms, and four families of pteridophytes (Figure S3; Tables S2 and S3).

Based on these pollen data, four climate parameters were estimated using a coexistence approach:[12] the mean annual temperature (MAT), the mean annual precipitation (MAP), 3HMP, and 3LMP (Figure S4 and Table S4). The curves of the median values of these climate parameters (Figure 1) are shown to represent the climate changes in central Tibet. The 3HMP and 3LMP were used here to constrain summer and winter precipitation, respectively.

The climate in central Tibet can be summarized by the MAT and MAP in the Lunpola Basin. The MAT fluctuated between 7.7°C and 15.6°C (Figure 1A). The MAT fluctuated three times during the period 26–20.6 Ma, initially increasing and then decreasing from 20.6 to 17.3 Ma, rising in 20.6–20.1 Ma, 19.6–18.8 Ma, and 17.9–17.7 Ma and decreasing in 20.1–19.6 Ma, 18.8–17.9 Ma, and 17.7–17.3 Ma. Finally, the MAT decreased from 17.3 to16 Ma.

The MAP fluctuated between 660 and 1,050 mm (Figure 1C) and fluctuated three times during the period 26–23.8 Ma; it decreased from 23.8 to 20.6 Ma and then increased initially and subsequently decreased during 20.6–16.7 Ma; it rose during 20.6–19.7 Ma, 19.6–19.1 Ma, and 18.5–17.7

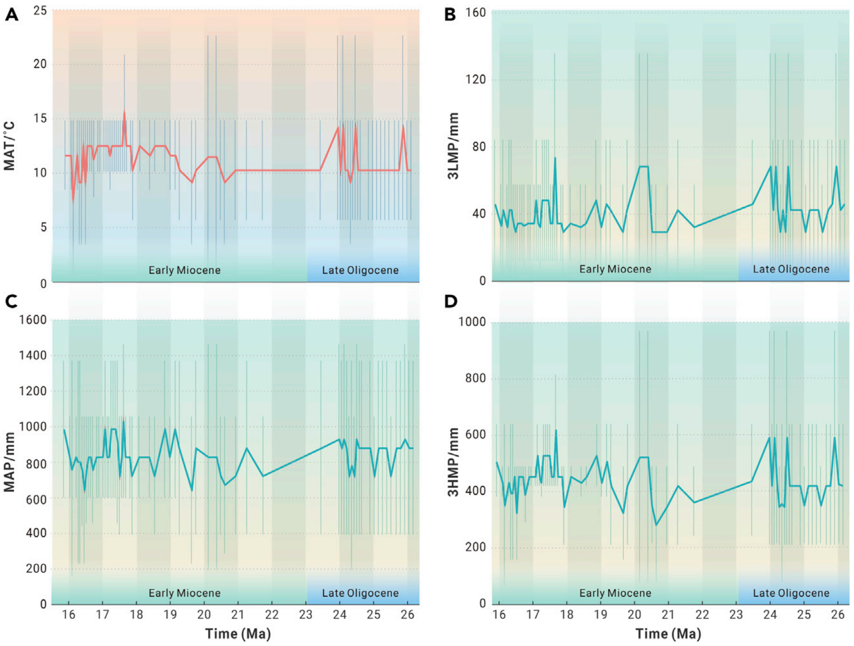

Figure 1. Climate data for central Tibet from 26 to 16 Ma
(A) Mean annual temperature (MAT, °C).
(B) Three consecutive lowest monthly precipitations (3LMP, mm).
(C) Mean annual precipitation (MAP, mm).
(D) Three consecutive highest monthly precipitations (3HMP, mm).
The vertical bars represent the ranges of the climate data in central Tibet, and the curves show the median values of these climate parameters.

Ma and then fell from 19.7 to 19.6 Ma, 19.1 to 18.5 Ma, and 17.7 to 16.7 Ma; and finally rose during 16.7–16 Ma.

These data indicate that the climate in central Tibet during the Late Oligocene to Early Miocene was warm and wet, i.e., the paleo-MAT was 9°C–17°C while the paleo-MAP was 350–740 mm higher than today's MAP[15] (Table S4).

DISCUSSION

Paleoclimate in the Lunpola Basin, central Tibet

To understand the central Tibet climate under global climate change, we compared the temperature (Figure 2) and precipitation (Figure S5) changes with those in central Europe[16] and the deep-sea temperature record[17] from the period 26–16 Ma.

During ca. 26–17.7 Ma, the temperature showed comparable fluctuations in central Tibet, central Europe,[16] and the deep sea[17] that included warming and cooling cycles (Figure 2). Two cooling events that corresponded to the Mi-1a and Mi-1b[18] glaciations, evidenced by the heavy oxygen isotopes present in the benthic foraminifera from the deep sea, were detected in central Tibet. These findings imply that the temperature in central Tibet and central Europe was strongly constrained by the global climate during this time interval. However, during ca. 17.7–16 Ma, in contrast to the deep-sea warming, cooling occurred in both central Europe and central Tibet (Figure 2); this event likely requires more quantitative evidence before it can be attributed to differences in the temperature change between the land and sea during this period.

The MAP showed a similar upward trend, with inconsistencies between central Tibet and central Europe for some time intervals[16] (Figure S5). These differences were likely caused by the different sources of precipitation mainly originating from the Indian Ocean and/or Pacific Ocean in central Tibet and from the Atlantic Ocean in central Europe.

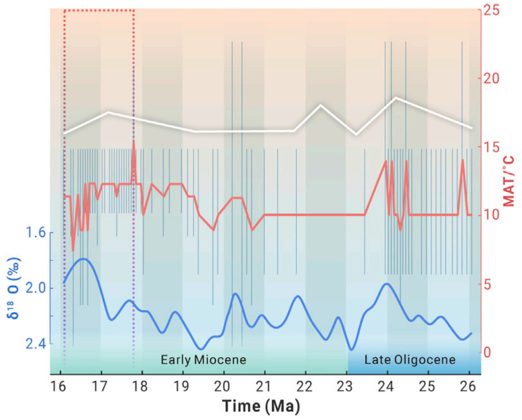

Figure 2. Temperature curves for central Tibet, central Europe, and the global deep sea during ca. 26–16 Ma The red line indicates the MAT (°C) in central Tibet; the white line records the MAT (°C) in central Europe, which was modified from Mosbrugger et al.;[16] the blue line displays the benthic foraminiferal oxygen-isotope curve (‰) in the deep sea, which was modified from Westerhold et al.[17]

The Asian monsoon occurred from 26 to 16 Ma

Here, we note that the annual precipitation range (AR: 3HMP minus 3LMP) (240–540 mm) and the proportion of 3HMP in the MAP (40%–60%) in the Lunpola Basin during ca. 26–16 Ma (Figure S6 and Table S4) fell within the range of those found in the modern Asian monsoon domains,[2] indicating that the paleomonsoon already existed. The modern Asian monsoon domains are defined by AR > 180 mm, i.e., summer (June to August) minus winter (December to February), and the proportion of summer precipitation >35% of the MAP.[2] In general, a larger AR value would correspond to a stronger Asian monsoon.[2] However, there is no way of separating the individual precipitation contributions of the East Asian monsoon and South Asian monsoon in this definition.

The precipitation in central Tibet was dominated by the paleo-Asian monsoon during 26–16 Ma, possibly because the plateau was farther south during that time period.[8,19] Today, westerlies control the modern climate in the Lunpola Basin. The Lunpola Basin in central Tibet is situated at 32.1° N and has obvious seasonality in the variations in surface air temperature and precipitation, i.e., cold and dry from December to February in winter and warm and rainy from June to August in summer.[15] According to the Land Climate Data of China (1951–1980),[15] published by the Information Department of Beijing Meteorological Center, the mean monthly temperature of Bange County where the Lunpola Basin (31°57′ N, 89°47′–89°49′ E) is located, is 7.5°C in summer and −10.1°C in winter, while the total precipitation is 224 mm in summer and 3.4 mm in winter (Meteorological Station No. 55279, 31°22′ N, 90°01′ E). The AR is 220.6 mm, while summer precipitation accounts for 72.7% of the MAP (308.3 mm). These precipitation data meet the criteria of a monsoon domain as defined by Wang and Ding,[2] i.e.,

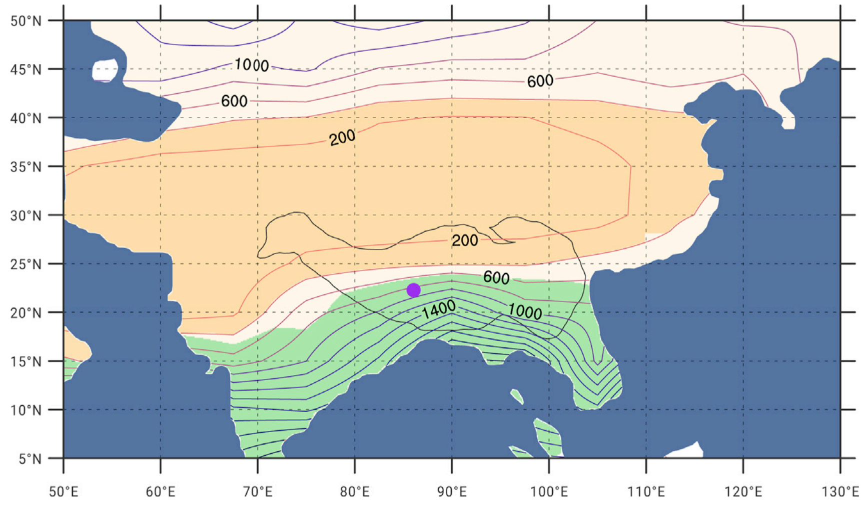

Figure 3. Simulated annual precipitation in the Pan-Tibetan plateau regions at 25 Ma The blue to orange lines display the simulated annual precipitation (mm); the green shaded area indicates the Asian monsoon domain; the yellow shaded area indicates the arid region; the black line is the topographic contour at 1,500 masl; the violet point is the studied section at 25 Ma. Please refer to Wang and Ding[2] and Liu et al.[8] for definitions of the monsoon domains and arid areas.

precipitation difference between summer and winter of >180 mm and the percentage of summer precipitation in the annual precipitation of >35%. However, since the MAP is less than 400 mm, the site now has a semi-arid monsoon-like climate.

Using paleoprecipitation data simulated with a climate model under the conditions of the reconstructed paleogeography, we found that the study area was located at ~22.27° N at 25 Ma, with an MAP of 857 mm (Figure 1C). By introducing the definition of the modern Asian monsoon domain[2] into this model, the data show that central Tibet belonged to the Asian monsoon domain, where the climate was controlled by the intertropical convergence zone (ITCZ) in summer (Figure 3) but not in winter. The ITCZ is a low-pressure zone where air flow from the Southern and Northern Hemispheres converges. The ITCZ moves north and south seasonally between 20° N and 20° S following the seasonal cycle of solar insolation, which is influenced by the position of the subsolar point, sea-land distribution, and topography. While its dominant areas range from 25° N to 25° S, the precipitation in these areas is far more abundant than that in the surrounding areas.[1] Recently, the tropical monsoon has been regarded as a product of the seasonal movement of the ITCZ between the Southern and Northern Hemispheres.[1] A monsoon climate with wet summers and dry winters can occur in areas that are controlled only in summer by the ITCZ. Central Tibet developed a monsoon climate during the Late Oligocene to the Early Miocene, likely under the influence of the ITCZ.

Strengthening of the monsoon from 20.6 to 16 Ma

Here, we notice that the AR trend first declined from 26 to 20.6 Ma and then increased during 20.6–16 Ma (Figure S6), indicating that the Asian monsoon in central Tibet first weakened and then strengthened.[2] If the monsoon in this region was solely controlled by the ITCZ, the intensity should have decreased as the Tibetan Plateau moved northward away from the ITCZ areas due to plate movement.[8] The unexpected strengthening of the Asian monsoon during 20.6–16 Ma seems to indicate that other driving forces also contributed to the evolution of the paleomonsoon.

The uplift of the Tibetan Plateau[20] likely coincided with plate movements[8,21] during this period, thereby enhancing the strength of the Asian monsoon. Central Tibet may not have reached its present elevation before the Middle Miocene,[13,19] implying that uplift occurred in this region before this time. In addition, some modeling studies have revealed that the uplift of the Tibetan Plateau/Himalayan Mountains would have enhanced the intensity and coverage of the Asian monsoon through mechanical dynamic effects[11] and/or heating effects.[10] At the same time, plate movement would also have led to changes in the land-sea distribution[21] in addition to the northward movement of the Tibetan Plateau, which might have increased the amount of monsoonal precipitation on the Tibetan Plateau by enhancing the temperature difference between land and sea.

Nevertheless, we have to admit that effects of uplift on precipitation are complex. The uplift probably not only led to an intensified monsoon and increased precipitation, but also enlarged the size of the plateau and increased the elevation of its southern flank. This could reduce the ability of the monsoon to carry water vapor over the mountains, consequently resulting in less precipitation eventually reaching central Tibet. More evidence is needed to understand the role of the uplift on the variations of monsoon during this period.

Previous research has already indicated that the uplift would change the temperature and precipitation on the plateau, based on modeling (e.g., Liu et al.[8]) and fossil evidence (e.g., Xie et al.[19]), although more solid data are needed to explore just how much change occurred.

It should be noted that the uplift process of central Tibet during 26–16 Ma is still hotly debated. For example, some researchers consider that the altitude of central Tibet already exceeded 4,000 masl at ~40 Ma based on oxygen-isotope paleoaltimetry[22] while others propose it was less than 2,300 masl[23] or 1,000 masl[24] in the Late Oligocene, based on fossil evidence. We estimated the elevation of the Lunpola paleolake in central Tibet to be 2,990–3,690 masl at ~24 Ma based on a *Marsilea* fossil and a pollen assemblage from the matrix of the same fossil bed, implying an uplift of 1,000–1,700 masl in central Tibet.[19] At the same time, we recognized distinct ecosystems that occurred along the southern slope, in the central region, and on the northern slope of the Tibetan Plateau during the Late Oligocene. The blocking of the Asian monsoon by the Tibetan Plateau probably contributed to the divergence of these ecosystems.[19]

Even so, the paleolatitudinal displacement may have had a greater impact on climate than elevational changes during 26–16 Ma according to our simulation results. In general, uplift leads to cooling, as shown in mountains today. We simulated the MAT at the study site at 25 Ma and today using a model, assuming that only elevation and latitude changed while other conditions remained constant. The simulation results show that the study site is presently at an altitude of ~4,800 masl and is located at 31.94° N with a 0.15°C MAT, while at 25 Ma it was at an elevation of ~3,800 masl and located at 22.27° N with a 16.9°C MAT. These results imply that a 16.75°C cooling occurred at the study site from 25 Ma to the present. If we take the lapse rate as 0.65°C/100 m, the elevation change would lead to a 6.5°C cooling from 25 Ma to the present. The remaining 10.25°C cooling can probably be attributed to the northward movement of the plate. Accordingly, we speculate that the climate change could be caused by the paleolatitudinal shift, together with uplift during this time period.

Orbital signals in the fluctuation cycles of the Asian monsoon

The curve of the annual precipitation ranges (3HMP minus 3LMP) shows the five weakening-strengthening cycles of the Asian monsoon (Figure 4). In detail, first, the Asian monsoon weakened during ca. 25.7–25.3 Ma and

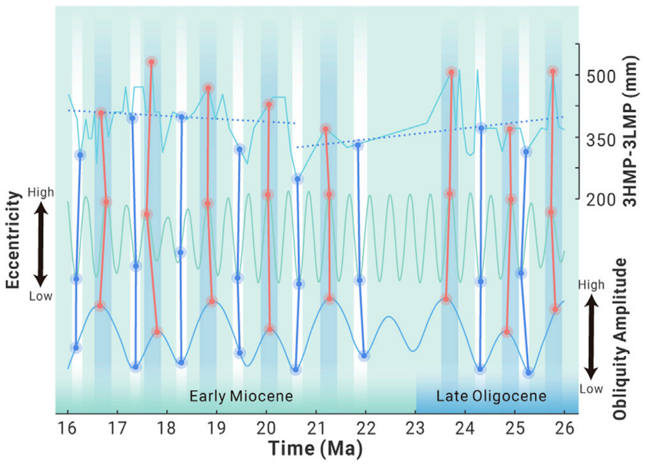

Figure 4. Intensity of the Asian monsoon in central Tibet during ca. 26–16 Ma The turquoise line indicates the AR (annual precipitation ranges, 3HMP minus 3LMP, mm) in central Tibet and was derived from this study; the blue dotted line illustrates the general trend of the AR; the green line indicates the fluctuations in eccentricity, which was modified from Ma et al.;[14] the blue line displays fluctuations in the maxima of the obliquity amplitude, which was modified from Boulila et al.;[26] the aqua bands and red vertical lines symbolize strong monsoons with high obliquity amplitude and eccentricity, while the white bands and blue vertical lines represent weak monsoons with low obliquity amplitude and eccentricity.

strengthened during ca. 25.3–24.3 Ma. Thereafter, the Asian monsoon weakened during ca. 21.6–20.6 Ma, ca. 20.1–19.6 Ma, ca. 18.8–17.9 Ma, and ca. 17.7–16.7 Ma, and subsequently strengthened during ca. 20.6–20.1 Ma, ca. 19.6–18.8 Ma, ca. 17.9–17.7 Ma, and ca. 16.7–16 Ma.

Spectral analysis at the 95% confidence level with a Monte Carlo test (Figure S7) revealed that the Asian monsoon fluctuated in ~1.35 Ma and ~0.33 Ma cycles, which appears to correspond to the eccentricity with a ~0.4 Ma cycle and the obliquity amplitude with a ~1.2 Ma cycle during ca. 26–16 Ma, as a strong/weak Asian monsoon is typically coupled with a high/low eccentricity and a maximum/minimum node of obliquity amplitude (Figure 4). Based on astronomical calculations[25] and sedimentary records,[26] previous studies have pointed out that in addition to ~41 ka cycles, obliquity also has ~1.2 Ma modulation cycles, which has significant impacts on global climate evolution on a million-year timescale. Previous research has typically considered tectonic movement as the main driving force of the Asian monsoon's evolution at the tectonic scale, namely the uplift of the Tibetan Plateau[20] and/or plate movements,[8,21] without considering long-period orbital forcings. The combination of factors proposed here would suggest that long-period orbital forcing also contributed to the evolution of the Asian monsoon on a million-year scale.

Orbital forcing has been regarded as an important force driving climatic changes in geological history.[27] Evidence has shown that ice sheets typically expanded in periods when the obliquity amplitude was low during the Cenozoic Era.[26] Zachos et al.[28] indicated that the Mi-1 glaciation during the Oligocene-Miocene transition was likely caused by the co-occurrence of low obliquity amplitude and eccentricity.

Obliquity is used to calibrate the position of the subsolar point on the Earth. A higher obliquity value is linked to a higher latitudinal position of the subsolar point, with more insolation being received by the high-latitude regions. Eccentricity is used to define the shape of the orbit of Earth. A higher value of eccentricity means that the Earth would receive more insolation at the perihelion, and vice versa.[27] Based on astronomical calculations and models, Matthews and Al-Husseini[29] suggested that glacial ice on Earth would have been melted by the high insolation received at the perihelion during high-eccentricity periods, while the ice would have expanded under the low insolation received at the perihelion during low-eccentricity periods.

The high obliquity in the maximal amplitude node together with the high eccentricity likely resulted in the strengthening of the Asian monsoon during the Late Oligocene to Early Miocene. On the one hand, the high eccentricity would have increased the total amount of solar radiation received by the Earth.[29] On the other hand, the high obliquity in the amplitude maximum node would have moved the subsolar point northward,[27] which may have triggered the northern movement of the ITCZ domain and thus strengthened the Asian monsoon in central Tibet and/or triggered ice melting and warming at high latitudes[29] by allocating more solar radiation energy to these regions. When the warming signals in high-latitude regions were transmitted to the middle- to low-latitude regions, the ITCZ domains would have shifted northward due to changes in the sea surface temperature gradient,[30] or the temperature difference between the land and sea would have increased due to the higher heat capacity of water than that of land. Thus, the Asian monsoon in central Tibet would have been strengthened.

REFERENCES

1. Gadgil, S. (2018). The monsoon system: land-sea breeze or the ITCZ? J. Earth Syst. Sci. ***127***, 1.
2. Wang, B., and Ding, Q. (2006). Changes in global monsoon precipitation over the past 56 years. Geophys. Res. Lett. ***33***, 272–288.
3. Wei, K., Ouyang, C., Duan, H., et al. (2020). Reflections on the catastrophic 2020 Yangtze river basin flooding in Southern China. Innovation ***1***, 100038.
4. Wang, Q., Spicer, R.A., Yang, J., et al. (2013). The Eocene climate of China, the early elevation of the Tibetan Plateau and the onset of the Asian Monsoon. Glob. Change Biol. ***19***, 3709–3728.
5. Licht, A., Van Cappelle, M., Abels, H., et al. (2014). Asian monsoons in a late Eocene greenhouse world. Nature ***513***, 501.
6. Miao, Y., Wu, F., Herrmann, M., et al. (2013). Late early oligocene East Asian summer monsoon in the NE Tibetan plateau: evidence from a palynological record from the Lanzhou basin, China. J. Asian Earth Sci. ***75***, 46–57.
7. Guo, Z.T., Ruddiman, W.F., Hao, Q.Z., et al. (2002). Onset of Asian desertification by 22 Myr ago inferred from loess deposits in China. Nature ***416***, 159.
8. Liu, X.D., DONG, B., YIN, Z.Y., et al. (2019). Continental drift, plateau uplift, and the evolutions of monsoon and arid regions in Asia, Africa, and Australia during the Cenozoic. Sci. China Earth Sci. ***49***, 1059–1081.
9. Spicer, R.A., Yang, J., Herman, A.B., et al. (2016). Asian Eocene monsoons as revealed by leaf architectural signatures. Earth Planet. Sci. Lett. ***449***, 61–68.
10. Wu, G., Liu, Y., He, B., et al. (2012). Thermal controls on the Asian summer monsoon. Sci. Rep. ***2***, 404.
11. Boos, W.R., and Kuang, Z. (2010). Dominant control of the South Asian monsoon by orographic insulation versus plateau heating. Nature ***463***, 218–222.
12. Mosbrugger, V., and Utescher, T. (1997). The Coexistence Approach—a method for quantitative reconstructions of Tertiary terrestrial palaeoclimate data using plant fossils. Palaeogeogr. Palaeoclimatol. Palaeoecol. ***134***, 61–86.
13. Deng, T., Wang, S.Q., Xie, G.P., et al. (2012). A mammalian fossil from the Dingqing Formation in the Lunpola Basin, northern Tibet, and its relevance to age and paleo-altimetry. Sci. Bull. ***57***, 261–269.
14. Ma, P., Wang, C., Meng, J., et al. (2017). Late Oligocene-Early Miocene evolution of the Lunpola Basin, central Tibetan Plateau, evidences from successive lacustrine records. Gondwana Res. ***48***, 224–236.
15. Information Department of Beijing Meteorological Center (1983). Land Climate Data of China (1951-1980) (China Meteorological Press).
16. Mosbrugger, V., Utescher, T., and Dilcher, D.L. (2005). Cenozoic continental climatic evolution of Central Europe. Proc. Natl. Acad. Sci. U S A ***102***, 14964–14969.
17. Westerhold, T., Marwan, N., Drury, A.J., et al. (2020). An astronomically dated record of Earth's climate and its predictability over the last 66 million years. Science ***369***, 1383–1387. https://doi.org/10.1126/science.aba6853.
18. Billups, K., Channell, J.E.T., and Zachos, J. (2002). Late oligocene to early miocene geochronology and paleoceanography from the subantarctic south Atlantic. Paleoceanography ***17***, 4-1–4-11.
19. Xie, G., Sun, B., Li, J.F., et al. (2021). Fossil evidence reveals uplift of the central Tibetan Plateau and differentiated ecosystems during the Late Oligocene. Sci. Bull. https://doi.org/10.1016/j.scib.2021.02.022.
20. Ruddiman, W.F. (2013). Tectonic Uplift and Climate Change (Springer Science & Business Media).
21. Ramstein, G., Fluteau, F., Besse, J., et al. (1997). Effect of orogeny, plate motion and land-sea distribution on Eurasian climate change over the past 30 million years. Nature ***386***, 788.
22. Rowley, D.B., and Currie, B.S. (2006). Palaeo-altimetry of the late Eocene to miocene Lunpola Basin, central Tibet. Nature ***439***, 677–681.
23. Su, T., Farnsworth, A., Spicer, R.A., et al. (2019). No high Tibetan plateau until the Neogene. Sci. Adv. ***5***, eaav2189.

24. Wu, F., Miao, D., Chang, M.M., et al. (2017). Fossil climbing perch and associated plant megafossils indicate a warm and wet central Tibet during the Late Oligocene. Sci. Rep. ***7***, 878.
25. Laskar, J., Robutel, P., Joutel, F., et al. (2004). A long-term numerical solution for the insolation quantities of the Earth. Astron. Astrophys. ***428***, 261–285.
26. Boulila, S., Galbrun, B., Miller, K.G., et al. (2011). On the origin of Cenozoic and Mesozoic "third-order" eustatic sequences. Earth Sci. Rev. ***109***, 94–112.
27. Ruddiman, W.F. (2007). Earth's Climate: Past and Future, Second edition (W.H. Freeman & Co Ltd).
28. Zachos, J.C., Shackleton, N.J., Revenaugh, J.S., et al. (2001). Climate response to orbital forcing across the oligocene-miocene boundary. Science ***292***, 274–278.
29. Matthews, R.K., and Al-Husseini, M.I. (2010). Orbital-forcing glacio-eustasy: a sequence-stratigraphic time scale. GeoArabia ***15***, 155–167.
30. Chiang, J.C.H., and Bitz, C.M. (2005). Influence of high latitude ice cover on the marine Intertropical Convergence Zone. Clim. Dyn. ***25***, 477–496.

ACKNOWLEDGMENTS

The authors thank Prof. Chun-Ju Huang from the China University of Geosciences (Wuhan) for a useful discussion. This research was supported by the Strategic Priority Research Program of the Chinese Academy of Sciences (no. XDB26000000), the China National Key Basic Research Program (2014CB954201), the National Natural Science Foundation of China (nos. 31570223, 41690115, and 32000174), the State Key Laboratory of Systematic and Evolutionary Botany (nos. LSEB2019-1 and LSEB2019-4), and the Chinese Academy of Sciences President's International Fellowship Initiative (2018VBA0016).

AUTHOR CONTRIBUTIONS

Y.-F.W., C.-S.L., and T.D. conceived the ideas; S.-Q.W and T.D. collected the samples; G.X. identified the pollen and spores and analyzed the data; G.X. and J.-F.L. searched the modern distributions and surface meteorological data of the pollen and spores; X.-D.L. simulated the paleogeography and paleoclimate by models; G.X., J.-F.L., Y.-F.Y., B.S., and S.-Q.W. performed statistical analysis; G.X. and Y.-F.W. wrote the first draft of this manuscript; D.K.F. rewrote some of the discussion and corrected the final manuscript; all authors contributed substantially to revisions.

DECLARATION OF INTERESTS

The authors declare no competing interests.

LEAD CONTACT WEBSITE

Yu-Fei Wang: http://people.ucas.edu.cn/~wangyufei.

Xiao-Dong Liu: https://publons.com/researcher/2784413/xiaodong-liu/. Tao-Deng:http://www.ivpp.cas.cn/sourcedb_ivpp_cas/zw/rck/yjy/200908/t20090811_2364079.html.

SUPPLEMENTAL INFORMATION

Supplemental information can be found online at https://doi.org/10.1016/j.xinn.2021.100110.

The Innovation, Volume *2*

Supplemental Information

Bridging the knowledge gap on the evolution of the Asian monsoon during 26–16 Ma

Gan Xie, Jin-Feng Li, Shi-Qi Wang, Yi-Feng Yao, Bin Sun, David K. Ferguson, Cheng-Sen Li, Tao Deng, Xiao-Dong Liu, and Yu-Fei Wang

This PDF file includes:

Supplemental Materials and Methods
Supplemental References 31-50
Figures S1 to S7
Table S1 and S3

Supplemental Materials and methods

Geological setting. The study section is the Dingqing Formation located in the Lunpori Mountains of the Lunpola Basin, Bange County, central Tibet (31°56′-31°57′ N, 89°47′-89°49′ E, 4650 m.a.s.l.) (see Fig. S1). This section is divided into 28 layers from bottom to top. For descriptions of the lithostratigraphy, see Deng et al.[13, 31]. Ninety-nine palynological samples were collected from layers 1 to 17 in this section (see Fig. S2).

Dating. Here, we adopt the concept of the stratigraphic sequence and identifying its age by Deng et al.[31]. Deng et al. reported a fossil rhino (*Plesiaceratherium*) from layer 17 in this section[13], which is the uppermost layer of sampling in this work. The genus *Plesiaceratherium* occurred worldwide in the late Early Miocene between the cold events Mi-1b at 17.8 Ma and Mi-2 at 16 Ma[13]. Because *Plesiaceratherium* was found together with a fossil hyena (*Percrocuta*) in Jiulongkou, Ci County, Hebei Province, China, which appeared later than the *Plesiaceratherium* in geologic history[13], we speculate that *Plesiaceratherium* appeared in China at ~16 Ma. At the same time, layer 7, which is a bentonite layer, was found to be 20.6 to 20.7 ± 0.1 Ma by LA-ICP-MS zircon U-Pb dating[32], while layer 2 was calibrated as 25.5±0.5 Ma by Su et al.[23] based on magnetostratigraphic data[33] and U-Pb dating[34]. Thus, we estimate the sedimentation rate of the upper part (layers 1-7) of this section as ~75 m/Ma and the bottom part

(layers 8-17) of this section as ~107 m/Ma; therefore, we can calculate the age of each collected sample (see Table S1). According to our calculations, the ages of our samples ranged from 26 to 16 Ma, covering the Late Oligocene to Early Miocene.

Pollen analysis. All the samples were treated with a heavy-liquid separation[35] method (density: 2.0 g/ml) to extract the pollen. The pollen and spores were identified as modern taxa using a Leica DM 2500 light microscope and by referring to the palynological literature[36-39] and monographs (Fig. S3). Abundant pollen and spores were found in 66 samples (56-1559 grains per slide), and over 300 grains were counted in most of these samples (Tables S2, S3). Those pollen and spores that could not be referred to modern taxa were regarded as an unknown type. Another 33 samples (Sample 12, 23-26, 28-37, 39, 40, 42, 44, 48, 50, 51, 54, 55, 60, 63, 65, 68, 81, 84, 94, 97 and 98) that preserved only a few pollen grains (2-26 grains per slide) were not counted or used in the reconstruction of the climatic data.

Paleo-climatic data. We applied the coexistence approach (CoA)[12] to reconstruct the climatic data in the Lunpola Basin during the Late Oligocene to Early Miocene. Four climate parameters were obtained (Table S4), i.e., MAT, MAP, 3HMP, and 3LMP. The modern distributions of pollen taxa in China[40] and the meteorological data within these distributions areas[15] were extracted to calibrate the climate interval of each parameter and the coexistence interval.

The detailed steps of calculating the climate parameters by the CoA are explained below. First, we ascertained which plant taxa lived in the Lunpola Basin, central Tibet,

during ~26-16 Ma based on the pollen extracted from sediments (see Table S2). Second, we obtained the distribution of each taxon in modern China by consulting Wu and Ding[40]. Third, we ascertained the climatic data for the regions in which each taxon lives from the Surface Meteorological Data of China (1951-1980)[15]. Next, we superimposed the climatic intervals of all the distribution areas of each taxon to constrain the range of the above four climate parameters. Finally, we calibrated the coincidence intervals of these climate parameters for all plant taxa (e.g., Fig. S4).

Paleo-monsoon. Here, we introduce the definition of the modern Asian monsoon[2] to calibrate the Asian monsoon during ca. 26-16 Ma. In the modern Asian monsoon domains, the annual range of precipitation (AR, summer minus winter) should be greater than 180 mm, the proportion of summer (June to August) precipitation in the annual precipitation should be greater than 35%, while larger values of AR correspond to stronger Asian monsoons[2]. We used the 3HMP and 3LMP to depict summer and winter precipitation, respectively; thus, the intensity of the Asian monsoon, as estimated by AR, could be described as 3HMP minus 3LMP. We also conducted spectral analysis to evaluate the frequency of the Asian monsoon intensity using the paleontological statistics software PAST 3.

Paleo-climatic modeling. The climate model used in this study was the Fast Met Office/UK Universities Simulator, which is a coupled atmosphere-ocean general circulation model (FAMOUS AOGCM)[41, 42]. The spatial resolution of the atmospheric component of FAMOUS was 5° × 7.5°, with 11 vertical layers, while the spatial resolution of the ocean component was 2.5° × 3.75°, with 20 vertical levels. The

atmospheric and oceanic components were coupled once every day, with no adjustments for fluxes. In the numerical experiment for the Late Oligocene (~25 Ma), we used a land-ocean configuration and topography that were reconstructed based on geological evidence. The experiment was run for 1000 years, and our analyses were based on the averages from the last 100 years. The land-ocean distributions during geological times were mainly based on the data from the GPlates database, an open-source software for the reconstruction of plate motions during the geologic time periods[43]. For Asia and Europe, the coastlines were modified according to varying regional geological evidence (e.g., Popov et al.[44]). The paleotopography and paleobathymetry were established based on previous reconstructions[45, 46] but revised using a large amount of published paleoelevation data (ref. Liu et al.[47]). The paleotopography of the Tibetan Plateau was reconstructed using paleoelevation data from various sources (e.g., Wang et al.[48]). Changes in paleolatitude were also considered for the Tibetan Plateau[49, 50]. More details on the experimental design and the boundary conditions related to the land-ocean configuration and the plateau topography can be found in Liu et al.[8, 47].

Supplemental References

31. Deng, T., Wang, X., Wu, F., Wang, Y., Li, Q., Wang, S. and Hou, S. Implications of vertebrate fossils for paleo-elevations of the Tibetan Plateau. *Global and planetary change*, (2019).
32. Mao, Z., Meng, Q., Fang, X., Zhang, T., Wu, F., Yang, Y., Zhang, W., Zan, J. and Tan, M. Recognition of tuffs in the middle-upper Dingqinghu Fm., Lunpola Basin, central Tibetan Plateau: Constraints on stratigraphic age and implications for paleoclimate. *Palaeogeography, Palaeoclimatology, Palaeoecology* **525**, 44-56 (2019).
33. Sun, J., Xu, Q., Liu, W., Zhang, Z., Xue, L. and Zhao, P. Palynological evidence for the latest Oligocene-early Miocene paleoelevation estimate in the Lunpola Basin, central Tibet. *Palaeogeography, Palaeoclimatology, Palaeoecology* **399**, 21-30 (2014).
34. He, H., Sun, J., Li, Q. and Zhu, R. New age determination of the Cenozoic Lunpola Basin,central Tibet. *Geological Magazine* **149**(1), 141-145 (2012).
35. Li, X. and Du, N. The acid-alkali-free analysis of Quaternary pollen. *Acta Botanica Sinica* **41**(7), 782-784 (1999).
36. Institute of Botany, C.A.S., *Sporae Pteridophytorum Sinicorum*. (Science Press, Beijing, 1976).
37. Institute of Botany, C.A.S., *Angiosperm Pollen Flora of Tropic and Subtropic China*. (Science Press, Beijing, 1982).
38. Wang, F.-X., Chien, N.-F., Zhang, Y.-L. and Yang, H.-Q., *Pollen Flora of China*. (Science Press, Beijing, ed. Second Edition, 1991).
39. Song, Z., *Fossil spore and pollen of China: The Late Cretaceous and Tertiary spore and pollen*. (Science Press, Beijing, 1999).
40. Wu, Z. and Ding, T., *Seed plants of China*. (Yunnan Science and Technology Press, Kunming, Yunnan, 1999).
41. Jones, C., Gregory, J., Thorpe, R., Cox, P., Murphy, J., Sexton, D. and Valdes, P. Systematic optimisation and climate simulation of FAMOUS, a fast version of HadCM3. *Climate Dynamics* **25**(2-3), 189-204 (2005).
42. Smith, R.S., Gregory, J.M. and Osprey, A. A description of the FAMOUS (version XDBUA) climate model and control run. *Geoscientific Model Development* **1**(1), 53-68 (2008).
43. Gurnis, M., Turner, M., Zahirovic, S., DiCaprio, L., Spasojevic, S., Müller, R.D., Boyden, J., Seton, M., Manea, V.C. and Bower, D.J. Plate tectonic reconstructions with continuously closing plates. *Computers & Geosciences* **38**(1), 35-42 (2012).
44. Popov, S.V., Shcherba, I.G., Ilyina, L.B., Nevesskaya, L.A., Paramonova, N.P., Khondkarian, S.O. and Magyar, I. Late Miocene to Pliocene palaeogeography of the Paratethys and its relation to the Mediterranean. *Palaeogeography, Palaeoclimatology, Palaeoecology* **238**(1-4), 91-106 (2006).
45. Herold, N., Seton, M., Müller, R., You, Y. and Huber, M. Middle Miocene tectonic boundary conditions for use in climate models. *Geochemistry, Geophysics, Geosystems* **9**(10), (2008).
46. Huber, M. and Goldner, A. Eocene monsoons. *Journal of Asian Earth Sciences* **44**, 3-23 (2012).
47. Liu, X., Dong, B., Yin, Z.Y., Smith, R.S. and Guo, Q. Continental drift and plateau uplift control origination and evolution of Asian and Australian monsoons. *Scientific Reports* **7**, 40344 (2017).
48. Wang, C., Dai, J., Zhao, X., Li, Y., Graham, S.A., He, D., Bo, R. and Meng, J. Outward-growth of the Tibetan Plateau during the Cenozoic: A review. *Tectonophysics* **621**(Complete), 1-43

(2014).
49. Chatterjee, S., Goswami, A. and Scotese, C.R. The longest voyage: tectonic, magmatic, and paleoclimatic evolution of the Indian plate during its northward flight from Gondwana to Asia. *Gondwana Research* **23**(1), 238-267 (2013).
50. Wei, H.H., Meng, Q.R., Ding, L. and Li, Z.Y. Tertiary evolution of the western Tarim basin, northwest China: A tectono-sedimentary response to northward indentation of the Pamir salient. *Tectonics* **32**(3), 558-575 (2013).

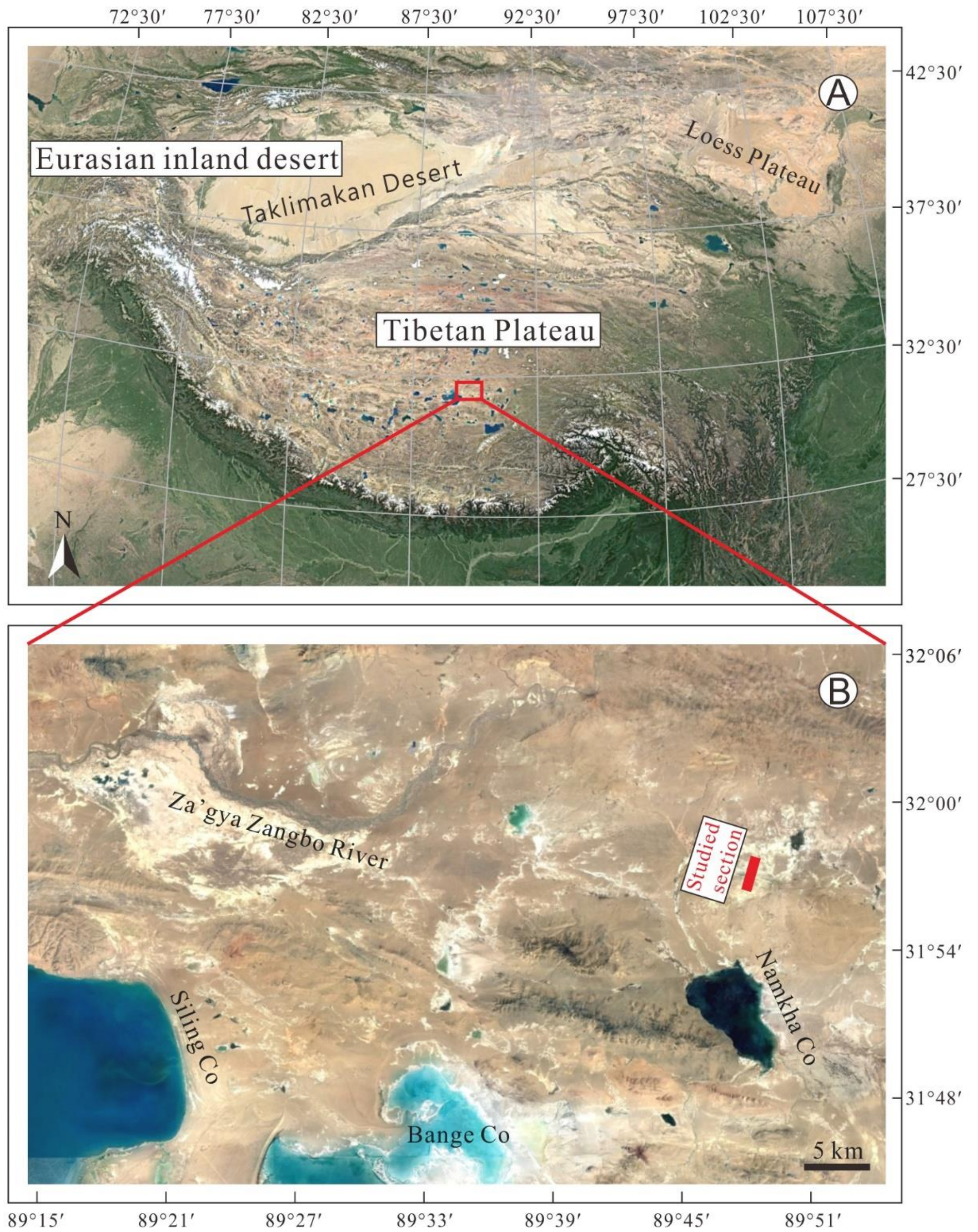

Fig. S1. Maps showing the position of the Lunpola locality (B) in central Tibet (A), modified from Google Earth images.

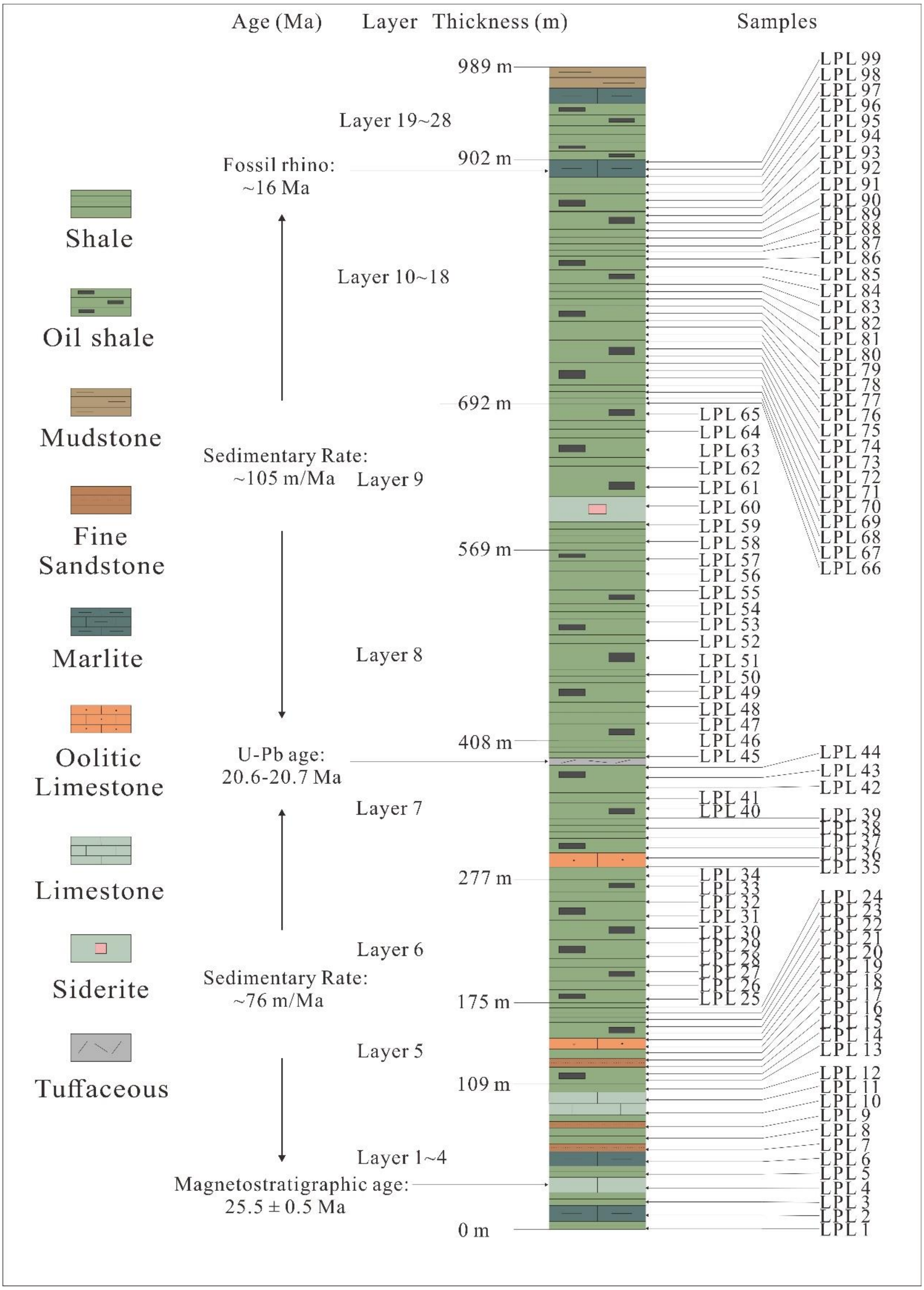

Fig. S2. Measured stratigraphical sequence of the Dingqing Formation section in the Lunpola Basin, central Tibet.

Age sources: fossil rhino, Deng et al.[13]; U-Pb age, Mao et al.[32]; magnetostratigraphic age, Su et al.[23].

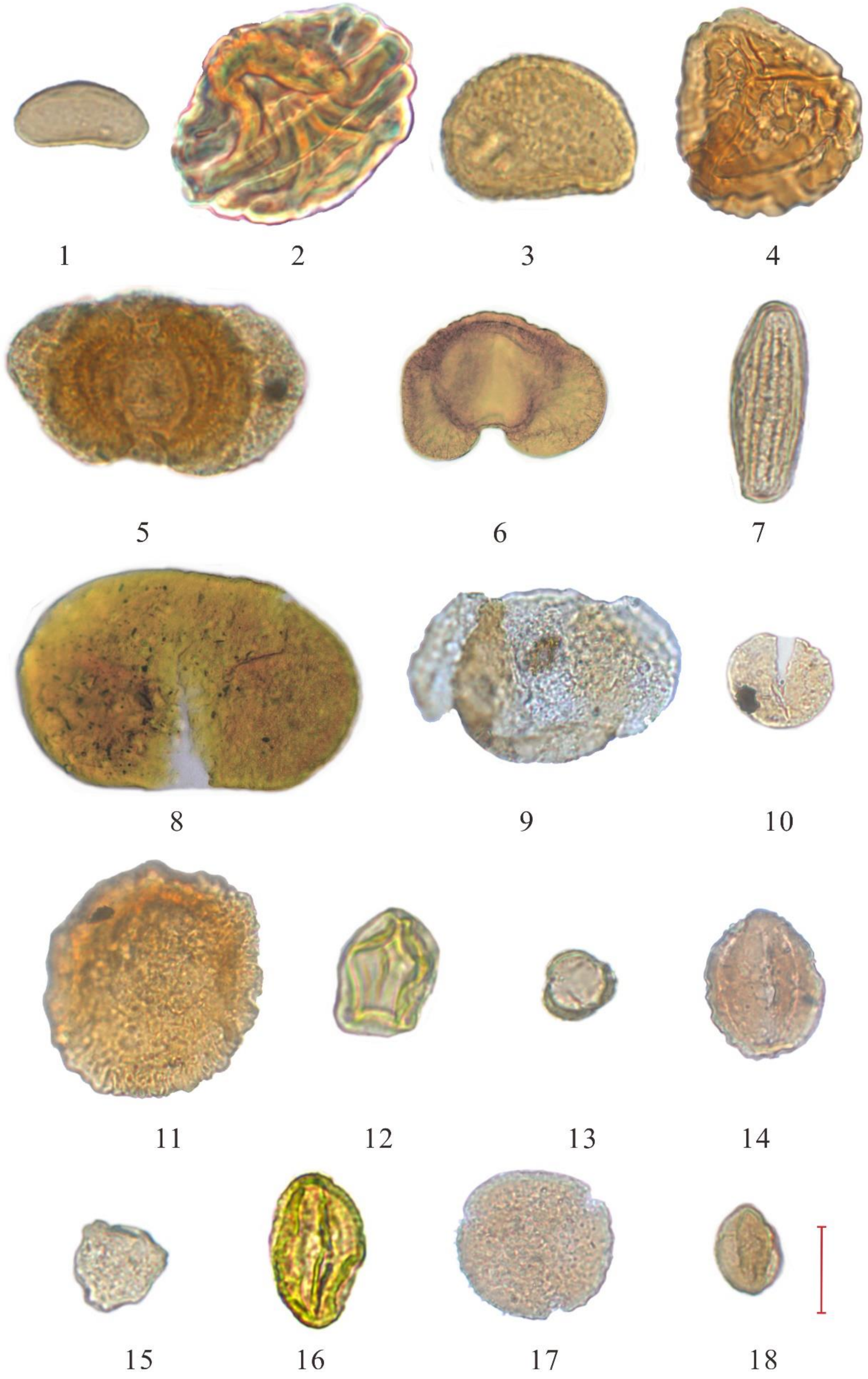
1
2
3
4
5
6
7
8
9
10
11
12
13
14
15
16
17
18

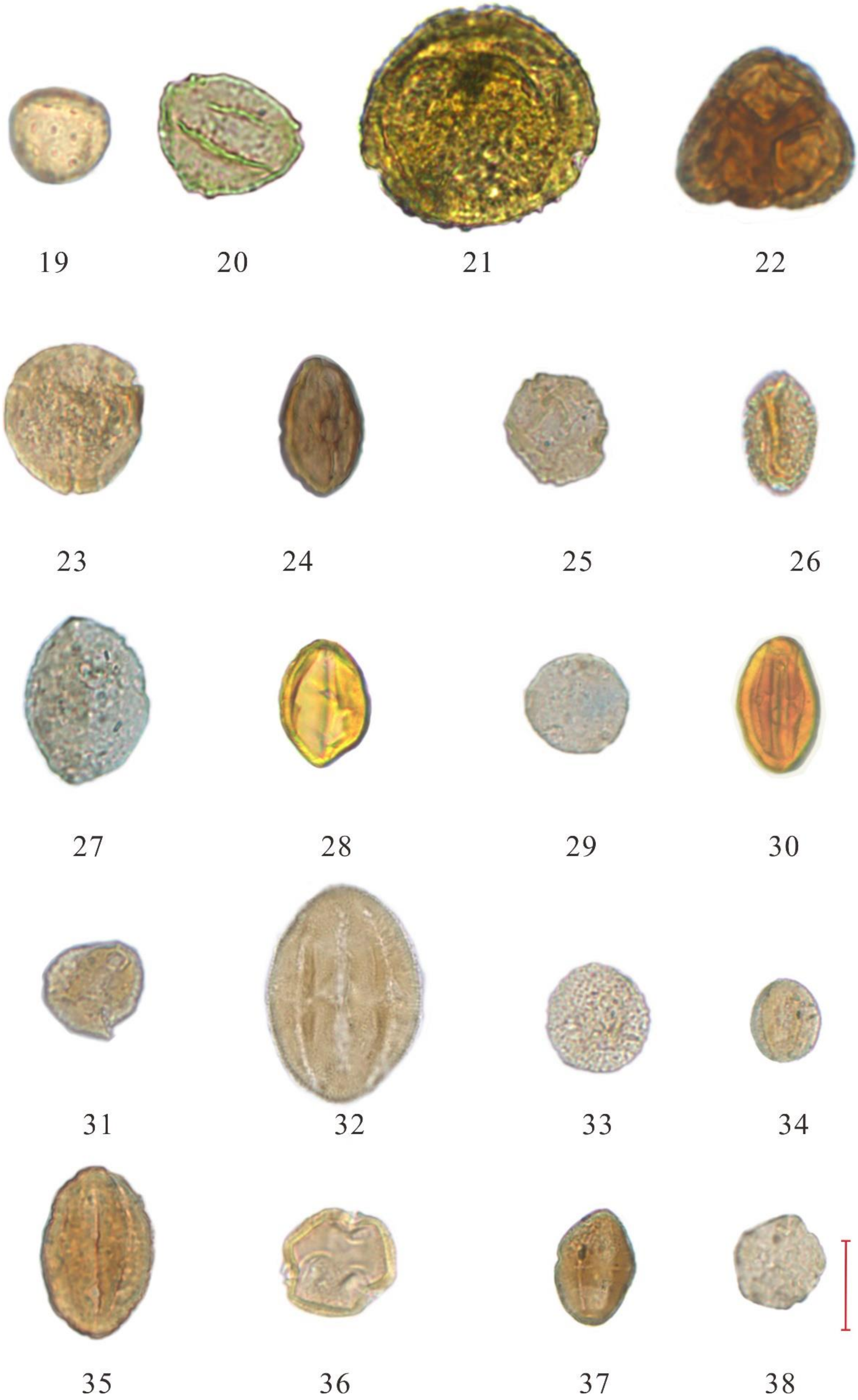

Fig. S3. Palynomorphs recovered from the Dingqing Formation. Scale bars: 20 μm
1. Athyriaceae; 2. Parkeriaceae; 3. Polypodiaceae; 4. Pteridaceae; 5. *Abies*; 6. *Cedrus*; 7. *Ephedra*; 8. *Picea*; 9. *Pinus*; 10. Taxodiaceae; 11. *Tsuga*; 12. *Alnus*; 13. *Artemisia*;

14. Asteraceae; 15. *Betula*; 16. Brassicaceae; 17. Caprifoliaceae; 18. *Castanopsis*; 19. Chenopodiaceae; 20. *Corylus*; 21. Dipsacaceae; 22. Ericaceae; 23. Euphorbiaceae; 24. Fabaceae; 25. *Juglans*; 26. Liliaceae; 27. Magnoliaceae; 28. Meliaceae; 29. Moraceae; 30. *Nitraria*; 31. Poaceae; 32. Polygonaceae; 33. Potamogetonaceae; 34. *Quercus*; 35. Ranunculaceae; 36. Rosaceae; 37. Rutaceae; 38. *Ulmus*.

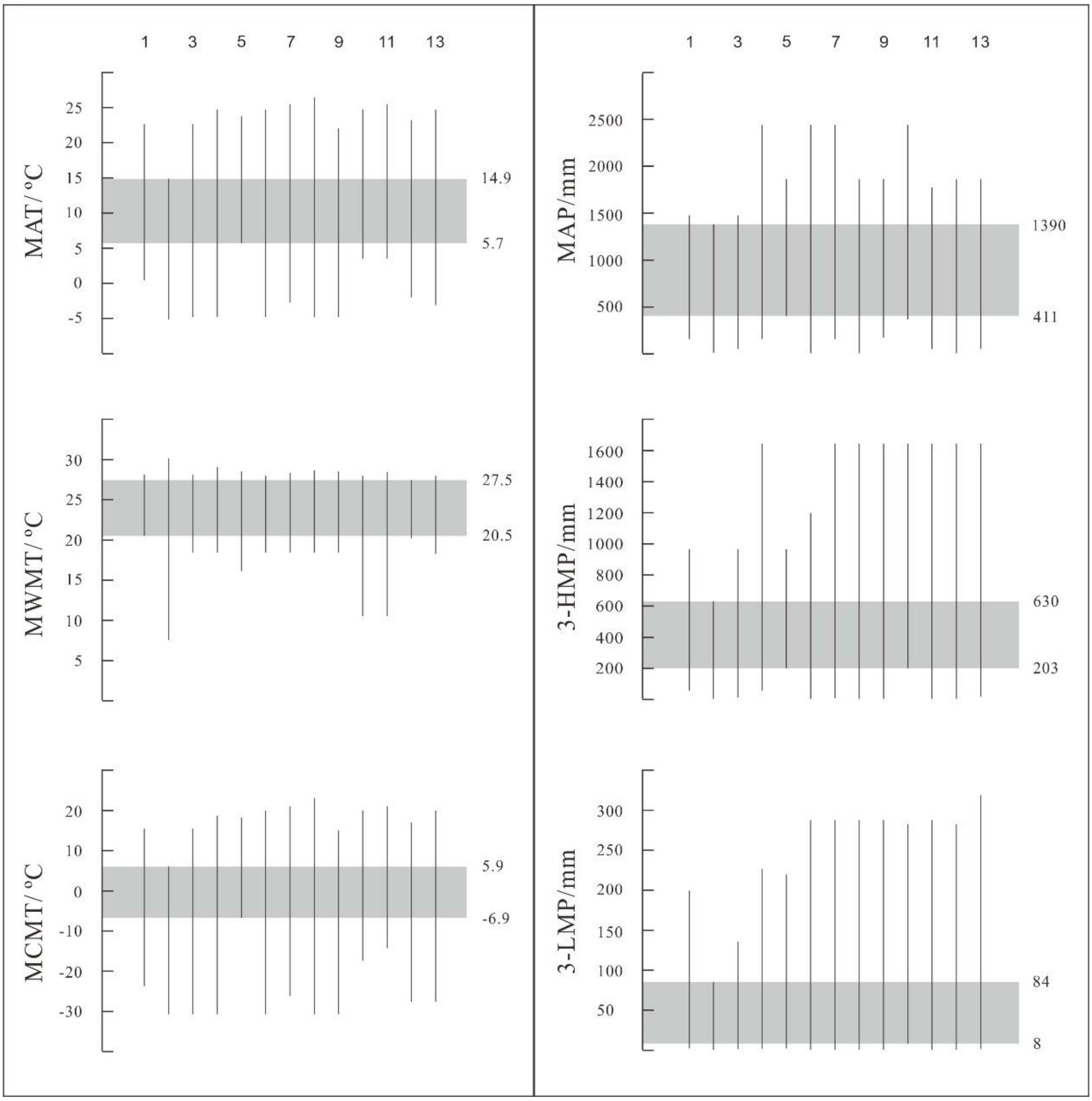

Fig. S4. The coexistence interval of the climatic parameters of the Sample No. 1 palynomorphs in Lunpola Basin

1. *Abies*; 2. *Ephedra*; 3. *Picea*; 4. *Pinus*; 5. *Tsuga*; 6. Chenopodiaceae; 7. Euphorbiaceae; 8. Fabaceae; 9. Lamiaceae; 10. Magnoliaceae; 11. Polygonaceae; 12. Ranunculaceae; 13. Rutaceae.

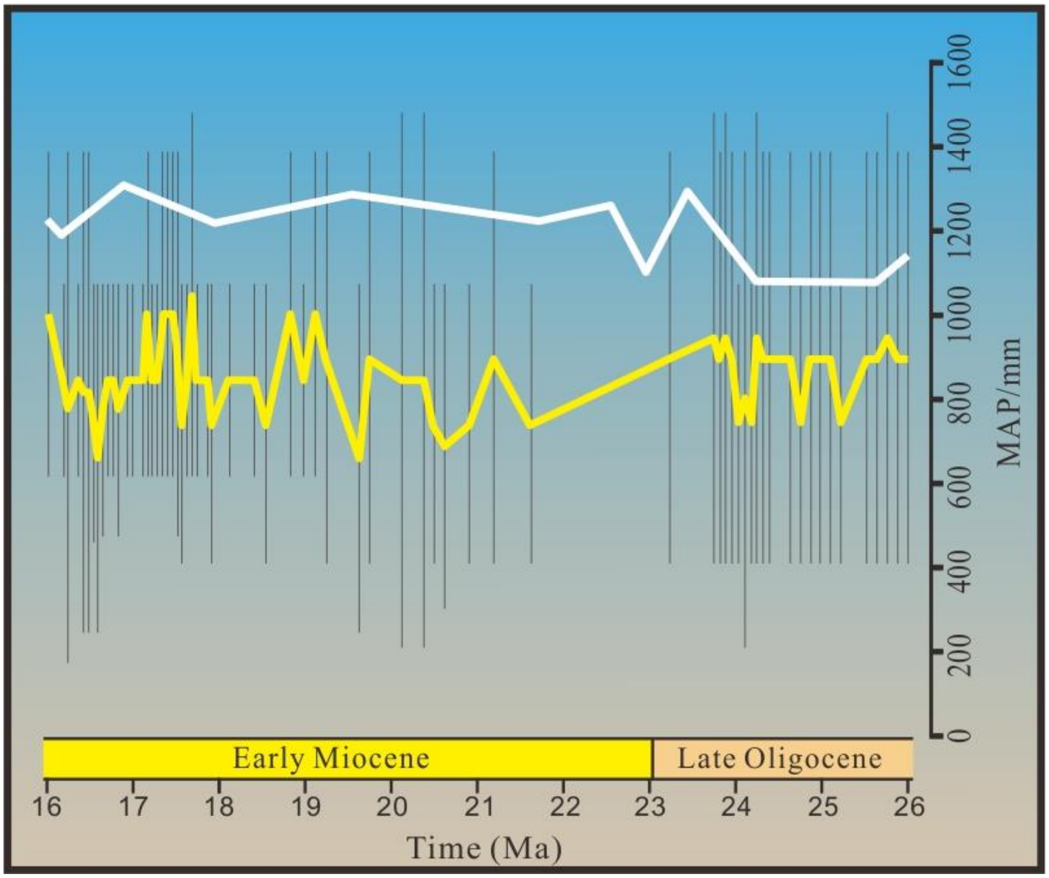

Fig. S5. MAP curves for central Tibet and central Europe ca. 26-16 Ma.
The yellow line shows the MAP (mm) in central Tibet, which was derived from this study; the white line indicates the MAP (mm) in central Europe, which was modified from Mosbrugger et al.[16].

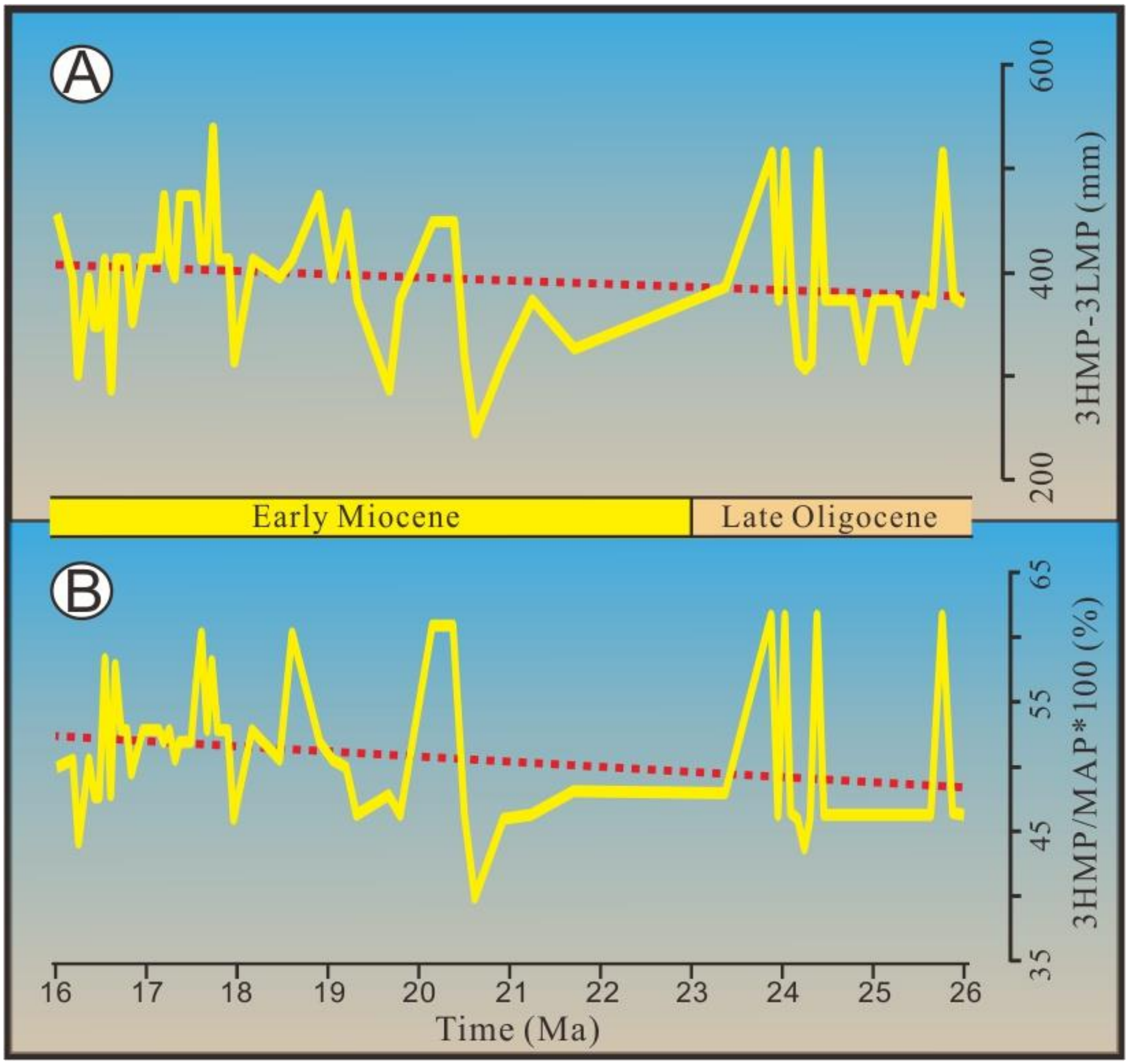

Fig. S6. Maps showing the fluctuations in the Asian monsoon in the Lunpola Basin, central Tibet during 26-16 Ma.
(A) the annual range of precipitation (AR, 3HMP minus 3LMP, mm); (B) the proportion of summer precipitation in the annual precipitation (%). The yellow line indicates the fluctuations; the red dotted line indicates the general trend.

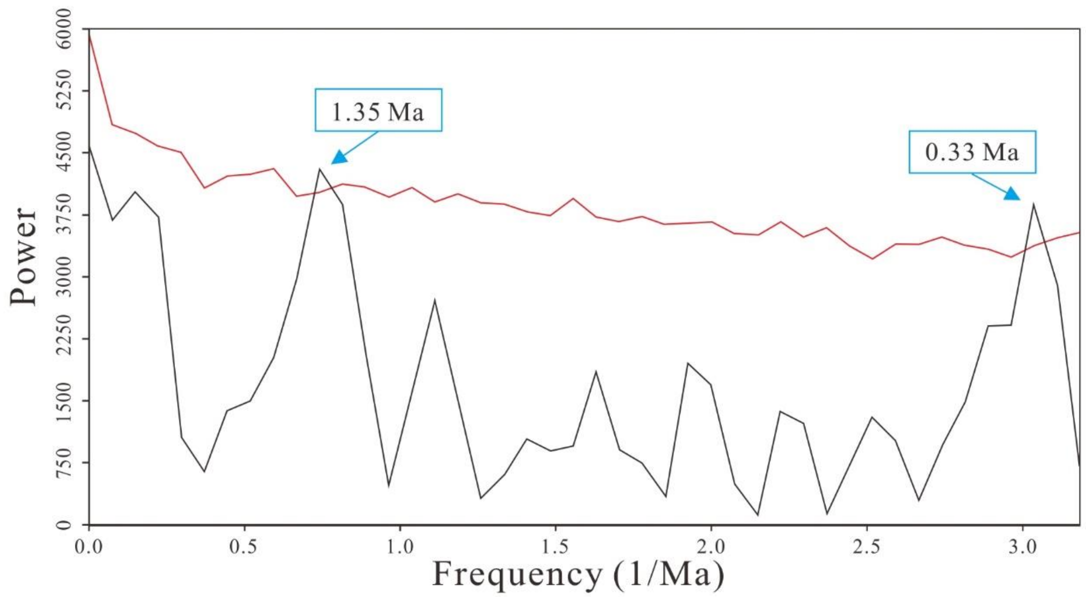

Fig. S7. Results of REDFIT spectral analysis.
The periodicity (from left to right): 1.35 Ma, 0.33 Ma; red line: significance line at the 95% confidence level for a Monte Carlo test

Table S1 Depth and calculated age of each sample

Layer	Thickness (m)	Amount of Samples	Sample	Depth (m)	Increasing of depth (m/sample)	Sedimentary Rate (m/Ma)	Increasing of time (Ma/sample)	Age (Ma)	Error range (Ma)
10-18	210	34	99	902.0	6.18	107	0.06	16.00	/
			98	895.8				16.06	± 0.1
			97	889.6				16.12	
			96	883.5				16.17	
			95	877.3				16.23	
			94	871.1				16.29	
			93	864.9				16.35	
			92	858.8				16.40	
			91	852.6				16.46	
			90	846.4				16.52	
			89	840.2				16.58	
			88	834.1				16.63	
			87	827.9				16.69	
			86	821.7				16.75	
			85	815.5				16.81	
			84	809.4				16.86	
			83	803.2				16.92	
			82	797.0				16.98	
			81	790.8				17.04	
			80	784.6				17.09	
			79	778.5				17.15	
			78	772.3				17.21	
			77	766.1				17.27	
			76	759.9				17.32	
			75	753.8				17.38	
			74	747.6				17.44	
			73	741.4				17.50	
			72	735.2				17.55	
			71	729.1				17.61	
			70	722.9				17.67	
			69	716.7				17.73	
			68	710.5				17.78	
			67	704.4				17.84	
			66	698.2				17.90	
9	123	8	65	692.0	15.38		0.14	17.96	
			64	676.6				18.10	
			63	661.3				18.24	
			62	645.9				18.38	
			61	630.5				18.53	
			60	615.1				18.67	
			59	599.8				18.81	

			58	584.4				18.96	
8	161	12	57	569.0	13.42		0.12	19.10	
			56	555.6				19.23	
			55	542.2				19.35	
			54	528.8				19.48	
			53	515.3				19.60	
			52	501.9				19.73	
			51	488.5				19.85	
			50	475.1				19.98	
			49	461.7				20.10	
			48	448.3				20.23	
			47	434.8				20.35	
			46	421.4				20.48	
7	131	12	45	408.0	10.92	75	0.15	20.60	± 0.1
			44	392.5				20.75	± 0.6
			43	381.6				20.89	
			42	370.7				21.04	
			41	359.8				21.18	
			40	348.8				21.33	
			39	337.9				21.47	
			38	327.0				21.62	
			37	316.1				21.77	
			36	305.2				21.91	
			35	294.3				22.06	
			34	283.3				22.20	
6	102	9	33	272.4	11.33		0.15	22.35	
			32	261.1				22.51	
			31	249.8				22.66	
			30	238.4				22.81	
			29	227.1				22.96	
			28	215.8				23.11	
			27	204.4				23.26	
			26	193.1				23.41	
			25	181.8				23.56	
5	66	12	24	170.4	5.50		0.07	23.64	
			23	164.9				23.71	
			22	159.4				23.78	
			21	153.9				23.86	
			20	148.4				23.93	
			19	142.9				24.00	
			18	137.4				24.08	
			17	131.9				24.15	
			16	126.4				24.22	
			15	120.9				24.30	
			14	115.4				24.37	

			13	109.9				24.44	
1-4	109	12	12	104.4	9.08		0.12	24.57	
			11	95.3				24.69	
			10	86.3				24.81	
			9	77.2				24.93	
			8	68.1				25.05	
			7	59.0				25.17	
			6	49.9				25.29	
			5	40.8				25.50	± 0.5
			4	31.8				25.62	± 0.6
			3	22.7				25.74	
			2	13.6				25.86	
			1	4.5				25.98	

Table S3. The palynomorph relative abundance in the Lunbori section

Palynomorph	RA(%)
Abies	4.4
Cedrus	0.5
Ephedra	3.0
Picea	37.0
Pinus	19.2
Taxodiaceae	/
Tsuga	0.9
Alnus	0.3
Artemisia	0.2
Asteraceae	1.0
Betula	0.8
Brassicaceae	/
Caprifoliaceae	0.1
Castanopsis	1.2
Chenopodiaceae	2.1
Corylus	/
Dipsacaceae	/
Ericaceae	0.1
Euphorbiaceae	0.2
Fabaceae	0.2
Fagaceae	1.3

Palynomorph	RA(%)
Juglans	0.1
Lamiaceae	0.6
Liliaceae	/
Magnoliaceae	/
Meliaceae	0.2
Moraceae	/
Nitraria	1.9
Poaceae	/
Polygonaceae	0.7
Potamogetonaceae	0.3
Quercus	0.4
Ranunculaceae	1.3
Rosaceae	0.4
Rutaceae	0.9
Ulmus	0.1
Athyriaceae	2.3
Parkeriaceae	0.1
Polypodiaceae	2.9
Pteridaceae	2.8
Unknown	12.4

/: less than 0.1%
